# Electrotunable Kapitza Resistance at Electrode-Water Interfaces: The Importance of Electrode Metallicity

**DOI:** 10.1021/acs.jpcc.6c00351

**Published:** 2026-05-07

**Authors:** Aidan Chapman, Fernando Bresme

**Affiliations:** Department of Chemistry, Molecular Sciences Research Hub, Imperial College London, W12 0BZ London, U.K.

## Abstract

The electrolyte-water-metal interface plays a vital role in electrochemical processes within nanocapacitors and in thermal management in nanoscale devices. Understanding the microscopic origins of thermal transport at these nanomaterial-fluid interfaces is crucial for advancing technologies in areas such as electrochemical energy storage and thermoplasmonics. Here, we use constant potential molecular dynamics simulations with fully dynamic electrodes to create steady heat fluxes in confined solutions that can respond to changes in the interfacial electrostatic environment at constant voltages. Our findings reveal that the Kapitza Resistance (KR) can be adjusted by applying voltage, altering ionic strength through the addition of salt, and, importantly, varying the metallicity of the electrodes. We show that the KR decreases with increasing electrode polarization, and salt concentrations above one molal further improve this voltage response, particularly with high effective metallicity (highly polarizable) electrodes. We attribute this response to a synergistic effect induced by the presence of the ions next to the electrodes and the reorientation of a nanometer-thick layer of water that solvates the electrodes. We present in our work a large-scale, nonequilibrium analysis that provides predictions of conditions necessary to tune the KR by considering experimentally relevant factors, including electrode metallicity, capacitance, and bias voltage.

## Graphical abstract

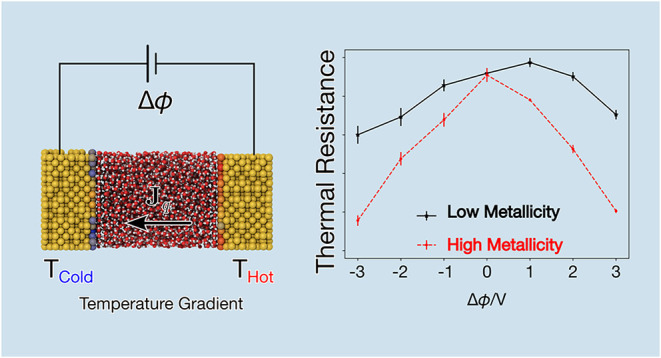

## Introduction

Heat transfer across electrode-fluid interfaces has become increasingly important, particularly in the context of interfacial electrode–solution double layers, which play a crucial role in the performance of batteries and supercapacitors.^1–3^ When voltage is applied, during the formation of the double layer heat is generated through both Joule heating and reversible heating mechanisms.^4–7^ Effective heat dissipation is essential, as elevated temperatures can negatively affect the longevity and performance of batteries and capacitors. This issue is particularly pronounced at small scales, where the thermal mass is lower.^8^

The Kapitza Resistance (KR) quantifies the heat flow resistance at interfaces during heat transport.^9–11^ When a heat flux is applied perpendicular to an interface, thermal resistance between materials and fluids results in a temperature discontinuity across the interface. Understanding the microscopic mechanisms that contribute to KR at nanomaterial-fluid interfaces is crucial for applications in nanofluids related to heat transport, as well as evaporation, condensation, thermoplasmonics, and nanoelectronics.^12–15^

Previous experimental studies have shown that hydrophilic interfaces exhibit lower KR compared to hydrophobic interfaces.^16^ These findings have prompted theoretical research aimed at identifying the microscopic factors that influence interfacial thermal transport mechanisms at these interfaces. Earlier research has examined various wettability conditions for substrate–fluid interfaces.^17–22^ Additionally, these theoretical investigations have revealed that the KR is dependent on the curvature of the interface.^19,23–25^

In batteries and capacitors operated at the device/macroscopic scale, heat dissipation is typically dominated by bulk conduction and contact resistances. In nanostructured or microscale electrochemical systems, where heat is generated and transported across nanometre-scale interfacial regions and pores, the Kapitza resistance can represent a substantial fraction of the local thermal resistance. There is indeed considerable interest in the community to deepen the understanding of nonequilibrium effects under nanoconfinement conditions, related to electrostatic fields (see refs 26,27 ). The use of electrostatic fields under nanoconfinement conditions can open up new opportunities in nanotribology^28^ and potentially in nanoscale thermal transport. Our focus is precisely this nanoscale regime and the electrotunability of interfacial heat transfer at metal–aqueous interfaces. The emerging opportunities to develop tunable KR strategies using electrostatic potential bias motivate our current study.

Computer simulations of charged nanoparticle surfaces have shown that increasing the charge magnitude^29^ results in a reduction of the KR. Similarly, these simulations revealed an enhancement of the heat transfer rate at charged metal-water interfaces.^30^ Moreover, it has been suggested that uniform electrostatic fields can influence the KR at water-material interfaces, especially under nanoconfinement conditions.^31–33^ Notably, the reduction in KR becomes significant at very high voltages, which, in some instances, may exceed the threshold voltage for water dissociation.^34^ These studies were conducted using nonpolarizable surfaces, highlighting the need to understand the conditions suitable for experimentally relevant setups.

Similarly, the KR of carbon and Room Temperature Ionic Liquids (RTILs) interfaces has recently been studied using both constant potential^35^ and constant charge methods.^36–38^ These studies indicate an inverse relationship between the KR and the applied bias voltage—a stronger applied potential difference enhances the interfacial thermal transport. However, there are no prior studies on interfacial heat transport that address the impact of electrode metallicity, an important property that influences the behavior of the metal-water interface.^39^

The complexity of modeling the electrode interface has led to significant advancements in the understanding of electrode–solution interfaces at a microscopic level, particularly in the context of constant potential computer simulations. Notable works include refs 26,40–49 . Additionally, critical discussions on this topic can be found in the literature (see refs 27 and 50). Constant Potential Methods (CPM) facilitate the simulation of electrode–solution interfaces under a constant electrostatic potential bias through continuous adjustment of the electrode charges to maintain the target potential. By design, they account for polarization effects, which are important for modeling charge-electrode interactions. Recent *ab initio* simulations have also demonstrated electronic spillover and linked this effect to high capacitances measured experimentally.^51,52^ The CPM and other polarizable methods have been extended recently to account for charge delocalization, spillover and quantum capacitance, without explicit inclusion of electronic degrees of freedom.^39,50,53–55^

In this work, we investigate metal-water interfaces through nonequilibrium simulations that incorporate both electrostatic potential bias and heat fluxes. We employ fully dynamic “live” metallic electrodes, allowing the atoms within the electrodes to fluctuate around their equilibrium positions, in contrast to keeping the electrode atoms frozen, as has been done in previous studies of electrodes using the CPM. This setup enables us to maintain the electrodes at two different temperatures and model the interfacial thermal transport process. Our approach enables an examination of the tunability of the KR in terms of electrode and solution compositions, as well as the electrostatic potential of electrolyte/water-metal interfaces. We specifically consider the electrode metallicity, a crucial factor in electrochemical processes. Our findings demonstrate significant changes in the KR with varying voltage, electrode metallicity, and ion concentration, with all these variables acting synergistically. We explore the double-layer interfacial structure and discuss the microscopic origins of this synergistic behavior.

## Methods

In this work, we employ the Constant Potential Method (CPM) with nuclei-centered Gaussians of width ∝ 1/η. This method does not resolve the electrode’s explicit electronic structure. Hence, phenomena like electron spillover into interfacial water and its significant contribution to the Helmholtz capacitance are not included explicitly. Recent DFT-based studies and analyses show that electronic response can substantially increase capacitance.^51,52^ Adjusting the Gaussian parameter η improves agreement with capacitance data, and it promotes ion adsorption (e.g., inner sphere Na^+^ at hollow sites^39^). In this work, we also employ the rigid model SPC/E, which removes high-frequency intramolecular modes. However, past work shows such modes contribute little to interfacial heat transfer at water–gold interfaces.^56^ Despite this, rigid water can under-represent polarization. While *ab initio* methods model electronic structure explicitly, at present they do not achieve the nanosecond sampling times, large cross-sectional simulation sizes, and simultaneous heat flux control under bias that we required. Therefore, we implemented dynamic electrodes with thermostats and finite-field CPM to maintain voltage while driving a stationary flux.

### Constant Potential Simulations

We employed the Constant Potential simulation Method (CPM)^40,41^ to model the electrode polarizability and the dependence of the KR with applied voltage. Figure 1 illustrates our simulation setup, which includes water or electrolyte solutions confined between the metallic electrodes.

**Figure 1:**
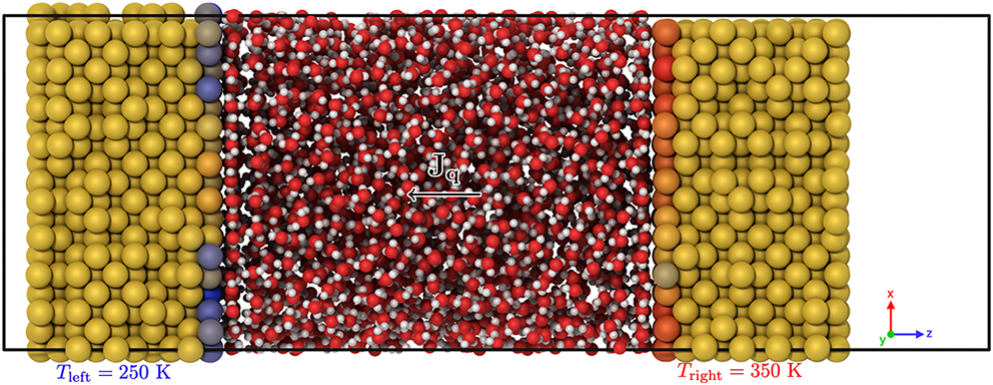
Simulation setup used in this study involves water or electrolyte aqueous solutions confined between charged metallic electrodes, with an imposed heat flux. The charge on the electrodes is adjusted using the CPM by altering the electrostatic potential. The atoms in the metallic electrodes are color-coded based on their charge: blue for –0.05 e, red for +0.05 e, and yellow for neutral atoms. The snapshot represents a configuration in which the distance between the electrode surfaces is approximately 50 Å, and the cross-sectional area of the electrodes is 1342 Å^2^. The potential difference across the electrodes is 2.0 V, and the electrodes are thermostated at temperatures of 250 and 350 K. The slabs are oriented with the (100) face in contact with the solution. The simulation cell is fully periodic, and the electrode–solution system is surrounded by a vacuum gap of approximately 20 Å.

We used a modified version of the LAMMPS^57^ implementation of the CPM.^45^ This approach allows for the charges on electrode atoms to vary, ensuring that the electrostatic potential remains constant on each atom within the electrode. As a result, the electrodes become polarizable, enabling them to respond to local changes in the double-layer structure. This is crucial for the interfacial systems studied here. By incorporating voltage as an explicit variable, the CPM approach establishes a direct connection to experiments, where voltage is typically controlled.

The CPM expands the standard phase space,^58^ which includes positions **r** ^*N*+*M*^ and momenta **p** ^*N*+*M*^, by incorporating the charges of the *N* electrode atoms, denoted as **q**. This results in a new set of dynamic variables, **x** = (**r** ^*N*+*M*^, **p** ^*N*+*M*^,**q**). During the simulation, these charges are subjected to constraints and are dynamically adjusted to produce the appropriate electrode potentials. The charges are adjusted instantaneously each time step, in a Born–Oppenheimer like approximation.^41^ The total charge on each electrode is enforced to be equal and opposite to retain system charge neutrality.

The atomic charges in the electrodes were modeled as Gaussian distributions,^40^ rather than as point charges. This approach results in a self-interaction among the electrode atoms, which introduces an energy penalty that is proportional to η during the charging process,^41,59^ where η controls the width of the Gaussian distribution. Each electrode atom contributes to the charge density at a given point in space **r**, according to the following expression^40,45^

1 $$\rho_{i}\left(r\right)=q_{i}{\left(\frac{\eta^{2}}{\pi }\right)}^{3/2}\exp\left\{-\eta^{2}|r-r_{i}{|}^{2}\right\}$$

where η has dimensions of inverse length, *q*_*i*_ is the total charge of the electrode atom *i*, which fluctuates during the simulation, and *r*_*i*_ is the position of the atom.

### Stationary Heat Flux and Constant Potential Simulations

In this work, we employ the CPM with fully dynamic electrodes that act as both heat sources and sinks. This configuration generates a steady heat flux while also adapting to changes in the electrostatic environment and local temperatures.

The electrostatic potential of the electrodes was established using the finite field variant of the CPM method, which allows for fluctuating electrode charges.^60^ The charges of the dynamic electrodes were calculated at every time step using the Conjugate Gradient (CG) method, which is implemented in the LAMMPS ELECTRODE package (for additional details, see the SI).^45^ This iterative numerical solver enables rapid calculation of the charges at each time step, making the simulation of truly dynamic electrode atoms computationally efficient. The use of the CG method with the finite-field method required modifications to the LAMMPS source code,^61^ as explained in the Supporting Section S2.1.

By enabling the electrode atoms to transfer heat to the electrolyte and accounting for both electrostatic and pair interactions with the electrolyte, we ensure that all contributions to the heat flux are included.

To generate the heat flux, we applied a Langevin thermostat^62^ with a damping time of 0.2 ps to each electrode. The water molecules and ions were integrated using the standard velocity-Verlet scheme and were not directly thermostated. The fluid exchanged heat only through interactions with the thermostated electrodes. Each thermostat was adjusted to a different predefined temperature to establish a temperature gradient across the system. At every time step, the net momentum of the system was removed.

The heat rate, *Q̇*, and the electrode cross-sectional area, *A*, defines the heat flux, *J*_*q*_

2 $$J_{q}=\frac{\mathrm{Q̇}}{A}$$

The Kapitza resistance is defined by the equation

3 $$R_{K}=\frac{\Delta T}{J_{q}}$$

where Δ*T* represents the temperature “jump” across the electrode–solution interface (see Figure 2, bottom). We discuss the calculation of Δ*T* in Section S4 of the SI. The heat rate is calculated from the time derivative of the total energy exchanged at each thermostat. The average of the two heat rates is then used to determine the overall heat rate. At steady state, the heat entering and leaving the two thermostats should be equal and opposite. Our method ensures good energy conservation for the parameters η and the time step of 2.0 fs used in this work (see Figure S2 in the SI).

**Figure 2:**
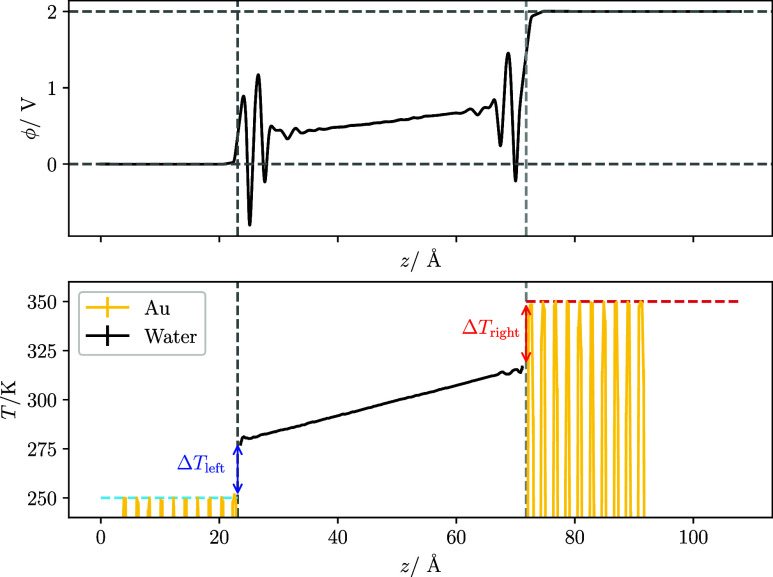
Spatial profiles of local properties—the electrostatic potential (top) and temperature (bottom). The system contains pure water and has a 2.0 V potential bias applied and electrode temperatures of (*T*_low_, *T*_high_) = (250 K, 350 K) for low (η = 1.81 Å^–1^, full line) metallicity electrode. The temperature and number density profiles are divided between two system components—the metallic electrodes (gold lines) and the water (black lines). The interfacial temperature jumps, indicated by Δ*T* in the bottom panel, are used to calculate the KR. See the main text for details.

The temperature profile needed to obtain Δ*T* were calculated by dividing the simulation box into planar slabs of thickness ∼0.2 Å along the surface-normal direction. The local temperature in each slab was computed from the kinetic energy of atoms whose centers lie in that slab, using

4 $$T\left(z\right)=\frac{2}{N_{\mathrm{dof}}k_{B}}\left\langle K\left(z\right)\right\rangle$$

where the number of degrees of freedom, *N*_dof_ = 3 for the ions and electrode atoms. For the rigid SPC/E water molecules we used the degrees of freedom derived in reference.^56^ The velocities were sampled every 10 ps and time-averaged over the full production trajectory. The resulting temperature profile was then fitted in the liquid region and in the electrode region using independent linear regressions (see SI Figure S3 for details on the fitting procedure), and these linear fits were subsequently employed to calculate Δ*T* and *R*_*K*_.

We note that the interfacial conductance is inherently temperature-dependent (as reflected in the different hot and cold Kapitza resistances reported in the Results and Discussion section). The Kaptiza resistance varies with the temperature of two phases in contact in nonisothermal systems, hence mimicking experimental conditions.

### Simulation Details

Our systems consisted of two fcc electrodes—with the (001) facet exposed—sandwiching a solution (see Figure 1). The solution in contact with the electrode consisted either of pure water or aqueous NaCl in one of two different molalities (1.00 mol/kg and 3.18 mol/kg), depending on the system being investigated. The simulations were performed with the SPC/E water model^63^ and the Dang force field for the ion–ion and ion–water interactions.^64^ The electrode-electrode interactions used the Lennard-Jones parameters for gold by Heinz et al.^65^ The Lennard–Jones description of the Au–water interaction used here follows similar models used in previous CPM studies. In our case, an explicit gold interaction is required to model heat transport within dynamic electrodes. We chose the Heinz et al.^65^ model, as it accurately describes gold–gold and gold–water interactions. We show in the Results and Discussion section that the models reproduce interfacial structural features consistent with earlier CPM and AIMD studies. Further simulation details are provided in the SI Section S1.

The pure water and 1 mol/kg NaCl systems contained 2160 water molecules, with an average water density of 0.997 g/cm^3^. The electrolyte portion of the system had the dimensions 36.63 × 36.63 Å^2^ and the initial length in the direction normal to the electrode plane as ≈ 50 Å. The electrode slabs each consisted of 1620 atoms in 10 layers. The resulting slabs had the dimensions 36.63 Å × 36.63 Å × 18.60 Å. Between the periodic images of the electrode, 20 Å of vacuum was added, and the total box size was 107.95 Å. The 1 mol/kg NaCl salt system used the same initial configuration as the water system, except for the addition of 39 ion pairs.

The 3.18 mol/kg NaCl system consisted of a box with the dimensions *L*_*x*_ = *L*_*y*_ = 48.79 Å and *L*_*z*_ = 85.91 Å. The electrolyte layer consisted of 4358 water molecules with 250 pairs of Na^+^ and Cl^–^ ions. Each electrode consisted of 4 layers of metal atoms arranged in an FCC structure, with the (001) facet exposed. The electrodes were placed ≈ 60 Å apart, with the electrolyte between. This system had 13 Å of vacuum between the periodic images of the electrodes. Prior to the addition of vacuum, the system was equilibrated at 300 K and 1 atm.

To maintain the interelectrode distance of ≈ 50 Å (60 Å in the 3.18 mol/kg system), the center of mass of each electrode was held in its original position using a harmonic restraint force, **f**_*r*_*e*__ = −*k*δ**r**, where δ**r** is the displacement of the center of mass of the electrode from its original position, and *k* is a force constant set to *k* = 50 kcal/Å^2^. The restraint was applied in all directions. We did not apply individual harmonic restraints to the gold atoms, because such restraints would alter the vibrational density of states and thermal conductivity of the solid slab, and could thereby affect the Kapitza resistance by perturbing the natural structure of the interface.

Each electrode was set to have a target potential of $\pm \frac{\Delta \phi }{2}$ , with Δϕ being the total potential drop across the electrodes. The left electrode (see Figure 1) was set to be negative, such that Δϕ = ϕ_right_ – ϕ_left_. Tables S2a,b, S4a,b, and S5a,b in the SI contain a summary of the simulations performed in this work, along with additional simulation parameters.

Each system was run for either 5 or 10 independent repeats, each lasting 1 ns for a total of 5 or 10 ns. The runs started from a well-aged system under a temperature gradient. The repeats used different thermostatting seeds and had an initial 400 ps of preproduction. All reported values, unless otherwise specified, represent averages computed from these repeats, with error bars reflecting the standard error of the mean from each set of repeats. Before data collection during the 1 ns production phase, all the systems were pre-equilibrated under bias for at least 1–2 ns, depending on the system, in order to establish stable density (see Figure S14) and temperature profiles.

Long-range electrostatics were computed using LAMMPS’ PPPM/electrode^66^ with accuracy 10^–7^, fully periodic 3D (tinfoil) boundary conditions, together with the finite-field^60^ constant-potential method. The electrodes were constrained to have equal and opposite total charges, preserving global charge neutrality. The electrolyte also contains equal numbers of cations and anions, so the full simulation cell remains globally neutral by construction. A vacuum gap (see Figure 1) was included only to suppress direct dispersion interactions between periodic images.

Regarding electrode charging (CPM), the constant-potential electrodes reach steady-state charge rapidly. Our Supporting Figure S4 shows that the characteristic charging times for pure water are 5 ps before plateauing. We checked that the electrode charge of the ionic solutions had converged before data collection.

As for the establishment of the thermal field, the time to form a stationary temperature profile across a gap of length *L* is $t_{\mathrm{th}}∼\frac{L^{2}}{\alpha }$ . For water at 300 K with thermal diffusivity α ∼ 10^–7^ m^2^/s, and *L* = 5 nm, *t_th_* ∼ 0.1 ns i.e., well below the 1 ns equilibration window (see also ref 67 for a convergence analysis of the transient thermal field in this geometry).

The equations of motion were integrated with a time step of 2.0 fs. We show in the results section that this time step predicts KR in agreement with previous studies using shorter timesteps.^56^

Further details of the simulation methods, including the implementation of dynamic electrodes for simultaneous simulations of polarization and thermal gradients, can be found in the Supporting Sections S1 and S2.

## Results and Discussion

We conducted our simulations using the constant-potential method^40,60^ to apply a potential difference, Δϕ, between the electrodes. We scan total electrode-to-electrode potential drops between −3 and 3 V to map trends. However, due to electrostatic screening and as discussed below, the electrostatic field inside the confined fluid is smaller than the field that would be obtained from this applied potential difference and the interelectrode distance. We also note that because classical force fields underpredict the double-layer capacitance, the applied Δϕ in CPM simulations does not correspond directly to an experimental electrode potential for a given surface charge.^68,69^ The system included two metallic “dynamic” electrodes that confined a fluid, either pure water or aqueous NaCl solutions. In addition to applying an electrostatic potential, we also introduced a heat flux by maintaining the electrodes at different temperatures (250 and 350 K).

Figure 1 shows a snapshot of the systems simulated in this work, and Figure 2 illustrates a typical profile of electrostatic potential and of temperature for a pure water system between two polarized electrodes. The target potential, ϕ_α_, of each electrode is set such that the potential difference between them is Δϕ = ϕ_*r*_–ϕ_*l*_, where α = *l*, *r* indicates the left or right electrodes.

The only tunable parameter in the CPM is the width of the Gaussian function used to model the atomic charges of the electrodes, denoted as η. This parameter is related to the metallicity of the surface and its susceptibility to polarization. In this study, we performed some computations using η = 1.81 Å^–1^. This value represents a low metallicity surface and is similar to the one used in the original calculations by Siepmann and Sprik.^40^ In that work, the parameter was fitted to reproduce the induced energy of the continuum theory and has been employed in recent studies of graphene-ionic liquid interfaces.^35,42^ We note that the metallicity parameter must be understood as an effective parameter to model the charge delocalization and not a complete description of the electrode electronic structure.

Serva et al.^39^ explored a range of ∼0.6 Å^–1^ to 1.8 Å^–1^ to account for various degrees of electrode metallicity, reporting significant changes in the differential capacitance with different η values. Therefore, in addition to the parameter mentioned above, we also conducted simulations using η = 0.88 Å^–1^, which is close to values used for modeling metallic surfaces.^39^ Hereafter, we refer to these values as Higher Effective Metallicity (HEM, more polarizable, η = 0.88 Å^–1^) and Lower Effective Metallicity (LEM, less polarizable, η = 1.81 Å^–1^).

The Kapitza resistance is defined by the ratio between the temperature discontinuity, Δ*T*, at the solid–liquid interface, and the heat flux, *J*_*q*_ perpendicular to the interface^11^

5 $$R_{K}=\frac{\Delta T}{J_{q}}$$

where Δ*T* represents the temperature “jump” across the electrode–solution interface (see Figure 2, bottom for an example).

### Pure Water

Figure 3 presents the interfacial density profiles of hydrogen atoms in water molecules. These profiles exhibit pronounced oscillations near the electrodes, with a distinct peak observed for oxygen atoms (see Figure S5b in the SI). Furthermore, the density distributions reveal a strong dependence on the local hydrogen structure adjacent to the electrode with not only voltage, but also the metallic character of the electrode. By contrast, the oxygen distribution is largely independent of the applied voltage (see Figure S5b in the SI). As discussed below, these findings suggest a modification of the orientational structure of water induced by the metallic properties of the electrode.

**Figure 3:**
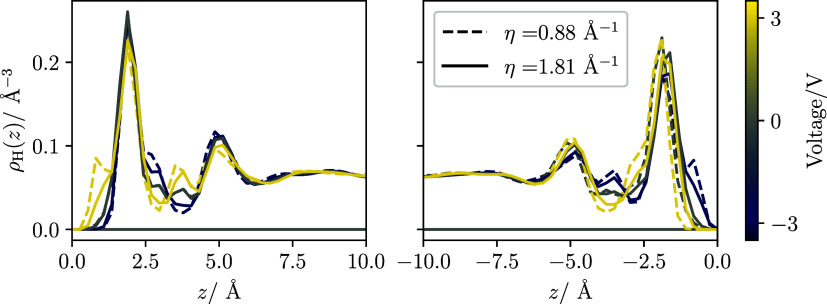
Hydrogen number density profiles of the pure water system simulated with thermostats set at 250 and 350 K and at different voltages. The metallicity parameter was set to LEM η = 1.81 Å^–1^ (full lines) or HEM η = 0.88 Å^–1^ (dashed lines). The left and right panels correspond to the density at the left (cold) and right (hot) electrodes, respectively. The *x*-axis indicates the distance from the metal-water interface plane. Oxygen density profiles for all voltages and η = 1.81 Å^–1^ are shown in Supporting Figure S5. The lines are color-coded by the imposed potential difference Δ*ϕ* to facilitate comparison between the two metallicities at the same applied bias. The corresponding surface charge densities are reported in Tables S2 of the SI.

The water density in the center of the confined region exhibits a slight decrease from the colder (left) side to the hotter (right) electrode (not shown). This variation is associated with the thermal expansion of the liquid in the thermal field. The density profile in the bulk region of the water remains unaffected by the applied voltage, indicating that the influence of electrostatic interactions is limited to the interfacial region, which extends to approximately ≈ 1 nm from the surfaces of the two electrodes.

In the stationary state, the system exhibits a constant heat flux and establishes a well-defined electrostatic potential, with an overall potential drop consistent with the applied voltage (see Figure S6 in the SI). The system reaches a steady charge quickly, within approximately 15 ps, with the longest characteristic charging time being τ = 2.7 ps (refer to Supporting Figure S4 for the charging dynamics during equilibration). The surface charge in our system ranges between ≈ −8 and ≈ +8 μC/cm^2^ for LEM and between ≈ −13 and ≈ +13 μC/cm^2^ for HEM (see Table S2a,b in the SI). These values are generally lower than those employed in previous simulation studies of KR at charged surfaces using a fixed surface charge method.^29^ The charge observed in our work is determined by the electrode metallicity parameter (η), the capacitance and the applied voltage.

Figure 2-top shows an example of the potential profile for the system under a voltage of 2 V. Further profiles for the other voltages can be seen in Supporting Figure S6. The system features potential drops at each electrode-water interface, along with strong oscillations in the first few layers of water and screening in the bulk. The potential drop at the electrode-water interface is greater between the negatively charged electrode and bulk water than the positively charged electrode. A similar asymmetry in the potential drop at the platinum-water interface has been reported in previous studies.^70^ The asymmetry arises from the different interactions that water molecules have with the two electrodes. For the negatively charged electrode, the hydrogen atoms of the water molecules approach the electrode more closely (see Figure 3), resulting in a larger potential drop. The rapid oscillations in potential observed near the interfaces, as also noted in reference,^70^ are due to the net orientation of molecules adsorbed at the interface. Advancing the discussion below, we note that the polarity and metallicity of the electrodes significantly influence the orientation of interfacial water molecules.

We calculated the interfacial electrostatic potential, δϕ, as the difference between the electrode potential and the potential measured at the second minimum in the oxygen density profile (see Willard et al.^70^ for a discussion of this approach), which we took to be 6 Å from the electrode surface. This method allows us to quantify the impact of the interface when a bulk potential cannot be defined. We note that choosing other nearby planes around the 6 Å position does not change qualitatively the trends of the computed capacitances. We also use this local reference for the confined electrolyte films, where the middle of the slit does not necessarily provide a well-defined bulk region in highly confined systems. The principal conclusions presented in this work (dependence of KR on voltage, metallicity and salt concentration) do not require a potential reference.

We also determined the Potential of Zero Charge (PZC), δϕ_PZC_, by identifying the root of the plot that depicts the relationship between interfacial potential and electrode surface charge (see Supporting Figure S8 for an example and Supporting Table S3 for values of the PZC).

Further, we calculated the interfacial differential capacitance of each electrode, $C_{D}=\frac{\partial \sigma }{\partial \Delta \delta \phi }$ , where Δδϕ = δϕ – δϕ_PZC_, either side of the PZC by linearly fitting the surface charge density as a function of Δδϕ. The Supporting Information contains Table S3 and Figure S9 which presents the full set of capacitances calculated and an illustrative example of the fitting procedure, respectively. Our capacitances range from 5 μF/cm^2^ to 10 μF/cm^2^ for LEM and from 10 μF/cm^2^ 20 μF/cm^2^ for HEM. The lower capacitances correspond to the positively charged electrodes. These calculations are consistent with values reported in previous studies, which reported capacitances: 5–8 μF/cm^2^ for the Pt-water interface,^70^ 4–5 μF/cm^2^ for the water-Au(001) interface using numerical differentiation,^68^ and 5 μF/cm^2^, using a fluctuation formula at 0 V, for the same water-Au(001) interface.^39^ ref 39 calculates the capacitance with a HEM surface (η = 0.86 Å^–1^) to be ≈ 7.5 μF/cm^2^, which is similar to the values obtained here. Our capacitance is similar to the 6.09 μF/cm^2^ value determined from semiclassical simulations by Andersson et al.^51^ These values are lower than those observed in experiments with gold electrodes.^71^ This discrepancy has been attributed to the significant contribution of the electronic degrees of freedom of the electrodes, causing effects such as electron spillover.^39,51^ When this electronic contribution is taken into account, the capacitance increases to approximately 60 μF/cm^2^.^51^ Notably, this elevated capacitance aligns with charge densities, ≈ 8 μC/cm^2^ similar to those considered in our study.^51^

Figure 4 presents the KR of the system, with both metallicities, as a function of the electrostatic potential difference between the electrodes. The temperatures of the two electrodes were maintained at *T*_cold_ = 250 K and *T*_hot_ = 350 K. At 0 V, the KR aligns with previously published calculations using the same gold-water model, including both nonpolarizable and polarizable surfaces (utilizing a core–shell oscillator model).^56^ Additionally, the KR values are comparable to those obtained using the DR-EAM image charge approach.^20^

**Figure 4:**
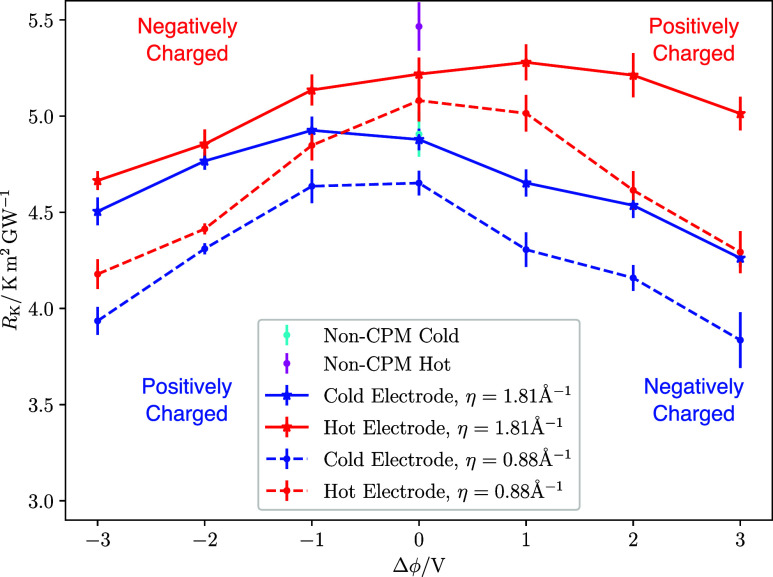
KR of the pure water-electrode system as a function of applied potential difference for two electrode metallicities, defined by the parameter η in eq 1. The cold electrode (blue lines) is set to 250 K and the hot electrode (red lines) is set to 350 K. The cyan (cold) and magenta (hot) points at 0 V represent the KR for a system with uncharged electrodes without the constant potential method. The potential difference represents the total drop between the hot and cold electrodes Δϕ = ϕ_hot_ – ϕ_cold_. For positive potential differences, the hot electrode is positively charged. Numerical data of the KR and charge density are provided in Table S2a,b in the SI. Because the hot and cold electrodes carry opposite surface charges at a given Δϕ, the extrema in KR do not occur at the same voltage. A comparison at equal surface charge density is provided in Figure 9. The labels in the figure indicate that the sign of the surface charge at positive and negative potentials depends on which electrode (hot or cold) is considered. Table S2 in the SI compiles data of Δ*ϕ* and the surface charge.

In Figure 4, we also present the results for the KR obtained from additional simulations using a nonpolarizable model that had the constant potential method deactivated, and the electrode charges fixed to 0. These results are compared with the CPM results at 0 V, where the electrode charge is very close to zero. The findings are very similar, suggesting that the polarization induced by the solvent on the electrode has a minimal effect on the KR of the metal-water interface at 0 V. Additionally, the similarity of the KR of LEM and HEM electrodes at 0 V further supports this conclusion. This similarity in KR between polarizable and nonpolarizable surfaces is consistent with previous works.^20,56^

The KR of water-metal interfaces can be adjusted by applying a voltage (see Figure 4). For the LEM electrode (η = 1.81 Å^–1^), the largest reduction of the KR, relative to its maximum at −1 V, is a significant ∼13.5% for an applied voltage of 3 V. For this potential, the electrode surface charge is ≈ 8.3 μC/cm^2^ (See Table S2a in the SI), and the total electrostatic field is 0.28 V/nm. These values are generally lower than those previously considered in simulations that maintain a constant charge^29^ or a constant electrostatic field.^32,33^ In our case, the electrode charge is influenced by both the capacitance of the interface and the metallicity of the electrode.

We conducted additional computations by increasing the electrode metallicity by reducing the parameter η in eq 1 to 0.88 Å^–1^. This increase in metallicity enhances the impact of the electrostatic potential on the KR, leading to a reduction of 23% when the voltage is raised to 3 V (see Figure 4, dashed lines).

The reduction of thermal resistance observed with higher metallicity is associated with the larger electrode capacitance (see Figure S9 and Table S3 in the SI). This larger capacitance results in an electrode surface charge that exceeds 10 μC/cm^2^ when subjected to an electrostatic potential of 3 V. These findings indicate that the relationship between KR and voltage is significantly influenced by the nature of the electrode. Specifically, greater metallicity leads to a more pronounced variation in resistance in response to changes in voltage.

Figure 4 also shows that the KR does not vary symmetrically around zero volts for LEM (solid lines), while it is primarily symmetrical for HEM (dashed lines). Advancing the discussion below, although Figure 4 is plotted as a function of the applied potential difference Δϕ, which is the control variable in the constant-potential simulations, the hot and cold electrode data become directly comparable when expressed in terms of the corresponding surface charge density. This is shown explicitly in Figure 9 (and its corresponding discussion), where the KR values collapse approximately onto a common charge-based trend. The most significant decrease in the KR occurs when water is in contact with the negatively charged electrode for both the cold (Δϕ > 0 V) and hot electrodes (Δϕ < 0 V). This observation suggests that water interacts more strongly with negatively charged electrodes, which aligns with the closer approach of the hydrogen atoms to the metallic surface as illustrated in Figure 3. Note that the oxygen density profiles feature very little change with voltage (see Figure S5), indicating minimal change in water adsorption at the interface.

For the LEM hot and cold electrodes (see Figure 4, solid lines), we observe a shift in the maximum KR from 0 to ∼1 V. A similar trend, where resistance increases from 0 V and then decreases for values greater than 1 V, is observed in constant potential simulations of graphene-RTIL capacitors.^35^ However, the KR for our metal-water interface is significantly lower, ∼5 m^2^ K/GW, compared to ∼20 m^2^ K/GW for carbon-RTIL interfaces. This indicates that interfacial thermal transport is much more favorable in metal-water interfaces.

The KR exhibits a significant dependence on electrode temperature (compare the red and blue lines in Figure 4), and it is lower for the cold electrode across all investigated voltages. This indicates that interfacial thermal transport across the electrode-water interface is more favorable at low temperatures. The lower first-peak in the oxygen density profile at the hot interface (see Figure S5 in the SI) indicates a slightly weaker adsorption to the surface. This is consistent with previous studies of graphene–water interfaces, showing a decrease in the KR with increasing adsorption.^72^

To gain a microscopic understanding of the behavior related to the KR, we calculated the Vibrational Density of States (VDoS) for the interfacial water molecules (those initially within 4.0 Å from the surface) and the surface atoms of the electrode (within the first layer). This type of analysis is commonly employed in the study of interfacial thermal transport.

Since we model water as a rigid molecule, the vibrational coupling between the electrode and water is influenced by changes in the orientation of water molecules near the interface, as well as the modification of the librational modes (see, e.g., ref 56). The methodology used to obtain the Vibrational Density of States (VDoS) is detailed in Section S9 of the SI.

Our calculations show that there is no significant change in the structure of the VDoS with increasing applied voltage (refer to Figure S15 in the SI). Consequently, the VDoS does not provide a consistent explanation for the microscopic origin of the KR reduction in our system. This conclusion aligns with the findings of Alosious et al.^35^ in their study of Room-Temperature Ionic Liquid (RTIL)-carbon interfaces.

The analysis of the interfacial structure of water (see Figure S5 in the SI) reveals a minor dependence of the oxygen density profile on voltage, suggesting that the electrostatic potential has a limited impact on water adsorption at the electrodes. In contrast, the hydrogen density profiles show significant variation (see Figure 3), with an increase in density next to the electrode when the electrode is negatively charged (ϕ > 0 V for the cold electrode and ϕ < 0 V for the hot electrode). This observation indicates a potential correlation between the dependence of the KR on voltage and the reorientation of interfacial water molecules as the voltage increases.

We computed angular distribution functions to quantify the changes in the orientational ordering of water molecules in contact with the electrodes (see Figure 5). Specifically, we calculated the orientational probability distribution function, *P*(θ), where θ is the angle between the bisector of the water molecule HOH (pointing from the oxygen toward the hydrogens) and the vector normal to the metallic electrode. This analysis was focused on water molecules with their oxygen atoms located within 4 Å of the hot electrode, approximately within the first water layer. A similar analysis for the cold surface is presented in the Supporting Section S10.

**Figure 5:**
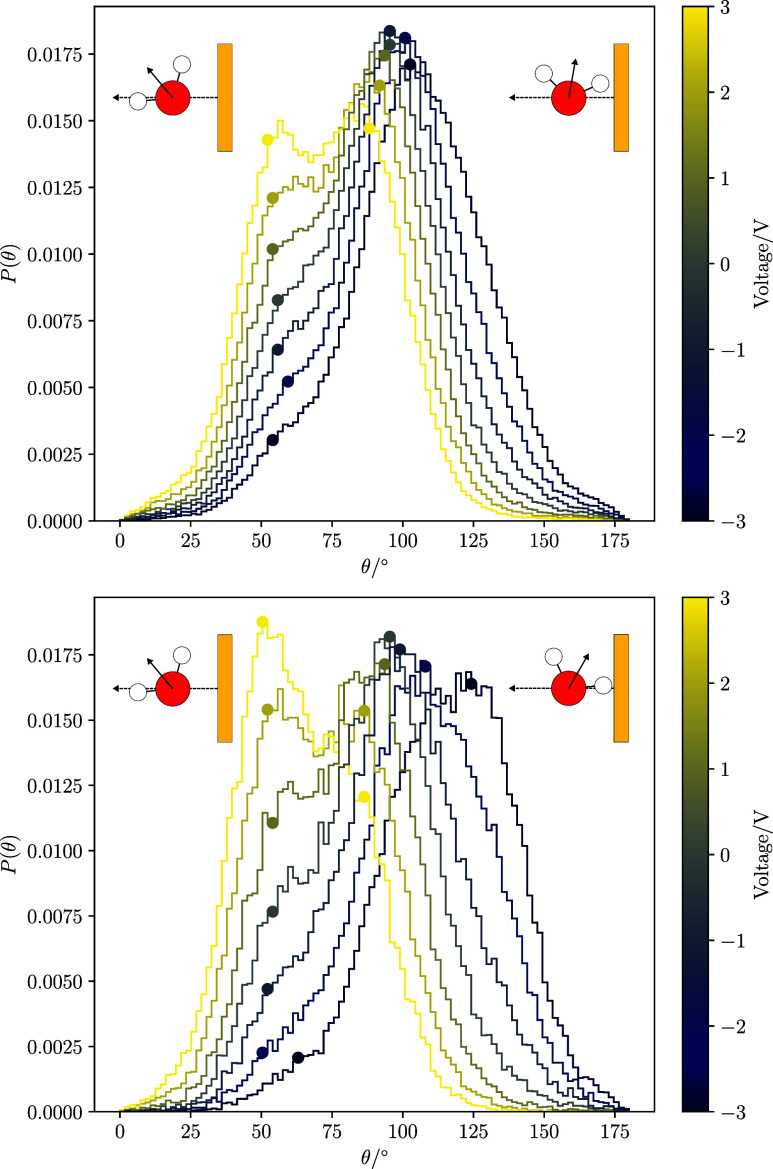
Normalized Distributions of the HOH angle bisector orientation in the first water layer at the hot interface. The selection of this layer is discussed in the main text. The angle θ is calculated between the dipole moment vector and the vector normal to the interface plane. The *y*-axis represents the probability density of observations of each angle. Each line is colored according to the potential difference between the two electrodes, Δ*ϕ* = ϕ_right_ – ϕ_left_. The top and bottom panels represent the results for LEM and HEM, respectively. The insets show sketches of the peak orientations at ± 3 V. The circles indicate the location of the maxima and shoulders in the probability distributions.

At zero applied potential difference, *P*(θ) displays a primary peak at approximately 100°, indicating that the water molecules are predominantly oriented perpendicular to the electrode plane, with a slight preference for the hydrogens to be directed toward the electrode (see insets in Figure 5). This tendency for water molecules to align flat against the interface is visually evident in simulation snapshots (see Figure S7). The snapshot also shows that water molecules tend to position themselves between the metal atoms in a square arrangement, a finding that is consistent with observations reported previously.^39,70^ At 0 V, the orientation distributions reveal a shoulder at approximately 50°. This shoulder, which becomes a maximum at higher voltages, corresponds to molecules with their oxygen atoms pointing toward the surface (see insets in Figure 5).

At highly positive voltages, the distribution becomes bimodal, with the shoulder peak evolving into a second population. The high-angle peak shifts to the left and exhibits a reduction in amplitude. When the surface is negatively polarized, the low-angle shoulder peak becomes less pronounced, and the high-angle peak shifts to the right. This shift suggests an increased tendency for the molecules to orient with their hydrogen atoms facing the interface.

Increasing the metallicity of the surface (η = 0.88 Å^–1^) heavily reduces the strong bimodal nature of the orientation distribution (see Figure 5, bottom panel) at the highest positive voltage. At 3 V, only a single peak at 50° is observed. At strongly negative potentials, the primary peak shifts from approximately 100° to approximately 120°. The stronger molecular polarization at the extremes of applied voltages is a result of the increased surface polarizability; the larger induced surface charges reinforce polarization. The locations of the peaks and bimodal nature we observe in Figure 5 are consistent with previous studies of the Au(001) interface, both classical^68^ and *ab initio* ^51^ MD studies. Similarly, the orientation we observe at high metallicity aligns qualitatively with the behavior noted in other *ab initio* simulations, which reported significant modification of the orientational structure of the molecules, with a trend to present a single peak probability distribution at high voltages.^73,74^

The reduction of the KR with increasing voltage is strongly correlated with greater orientational ordering, driven by the enhanced surface charge at both positive and negative electrodes under high voltages. However, the voltage dependence of the KR is asymmetric about 0 V for the LEM electrode (Figure 4, solid lines). Our results suggest a competition between two dominant molecular orientations; the relative population of each varies with the applied voltage. When the LEM electrode is positively polarized, the orientation distribution is bimodal. Molecules with oxygen pointing toward the electrode only appear significantly at 1.0 V, coinciding with the KR maximum in Figure 4 (solid lines). In contrast, at zero and negative polarization, water molecules predominantly orient with hydrogens toward the interface. This difference in orientation under polarization explains the observed KR asymmetry (Figure 4).

As metallicity increases, a dominant orientation becomes more favorable, reducing the KR difference between positively and negatively charged electrodes. Consequently, KR decreases symmetrically around 0 V as voltage varies (Figure 4, dashed lines). Analysis of the cold electrode shows similar orientational ordering to the hot interface, mirrored to account for the opposite surface polarization (see Supporting Section S10). The additional KR reduction for highly metallic surfaces is attributed to their greater induced charges (Table S2a,b in the SI). The higher degree of orientational ordering results from the more polarized surface, which enhances thermal transport across the interface.

### Electrolyte Solutions

Applying a voltage across electrodes can significantly alter the interfacial structure of electrolyte solutions in contact with them. In this section, we will explore how voltage and salt concentration (1 mol/kg, 3.18 mol/kg) influence the KR of sodium chloride (NaCl) aqueous solutions. We focus first on the 1 mol/kg NaCl system.

Figure 6a,b display the number density profiles of ions in a 1 mol/kg aqueous solution subjected to an applied voltage and heat flux. The left and right electrodes were maintained at temperatures of 250 and 350 K, respectively. The profiles indicate a pronounced accumulation of counterions as the voltage increases, with significant counterion condensation occurring at the electrode interface when the potential difference reaches 3 V (see the left side of Figure 6a or the right side of Figure 6b). Similar to the pure water system, the density profile of the oxygen atoms shows only minor changes with voltage (refer to Figure S10 in the SI).

**Figure 6:**
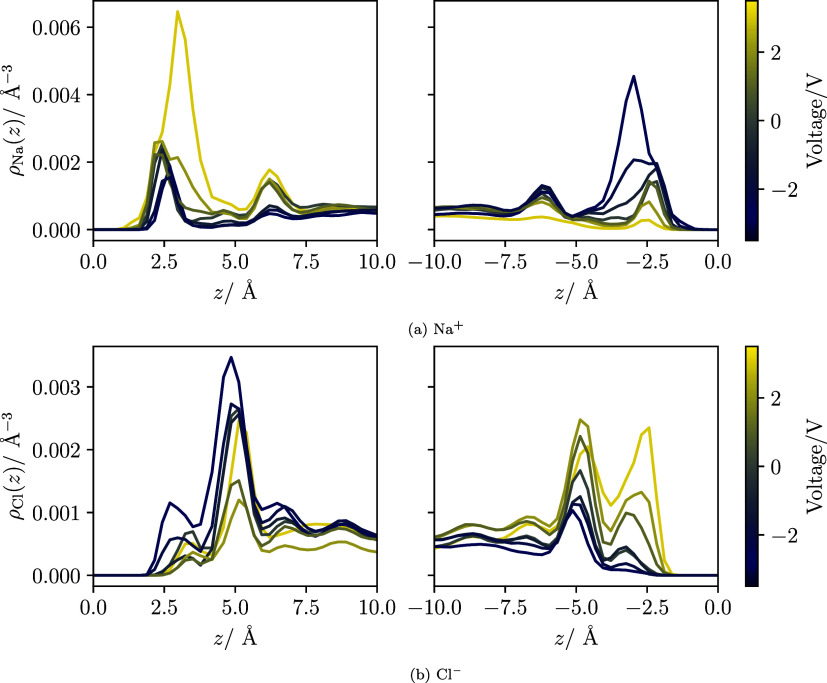
Density profiles of the ions (a) Na^+^ and (b) Cl^–^ in the 1 mol/kg NaCl system for several applied voltages and (*T*_low_, *T*_high_) = (250 K, 350 K). The line color indicates the voltage applied between the electrodes. A positive voltage corresponds to the left/right electrodes being negatively/positively polarized. All the results were obtained with a metallicity corresponding to η = 1.81 Å^–1^. These voltage-resolved ion density profiles provide the quantitative basis for the interfacial structural motifs later illustrated in Figure 8. Table S4 in the SI compiles data of Δϕ and the surface charge.

Compared to the pure water system, the electrostatic potential profile (Figure S12 in the SI) displays almost no slope within the interior of the film, reflecting the strong screening effects of the electrodes, due to the presence of counterions and the formation of the electric double layer. The oscillatory behavior of the electrostatic potential profile (see Figure S12 in the SI) indicates a significant alteration of the electrostatic field near the electrodes, due to the orientation of the water molecules. These oscillations are similar to those found in pure water (see Figure 2 and Supporting Figure S6). Like pure water, the electrostatic potential of the electrolyte solution exhibits a greater potential drop near the positively charged electrode, regardless of the electrode temperature. This observation is connected to the differing differential capacitances of the positive and negative electrodes, which have typical values of 5 to 10 or 8 to 20 μF/cm^2^, for LEM and HEM, depending on whether the potential is below or above the PZC (see Table S3 in the SI). For comparable polarizations, the differences between the capacitances of the hot and cold electrodes are minimal. Thus, the electrode temperature appears to play a secondary role in determining the capacitance.

Figure 7a illustrates the relationship between the KR and voltage for the 1 mol/kg NaCl solution, considering two values of the metallicity parameter η = 1.81 Å^–1^ and 0.88 Å^–1^. As in the pure-water case, Δϕ is used because it is the external control variable. The corresponding comparison at equal surface charge density is provided in Figure 9, which shows that the KR trends for hot and cold electrodes are largely unified when represented in terms of electrode charge. In Figure S13 of the SI, we present the example temperature profiles for the systems with η = 0.88 Å^–1^. At zero volts, the KR is comparable to that observed in pure water. Similarly, the resistance is also lower for the cold electrode, indicating more favorable heat transport at low temperatures. The KR shows significant changes with increasing voltage. A potential difference of 3 V results in a reduction of 14–19% for low metallicity and around 30% for high metallicity. These reductions are more significant than those observed for pure water. This observation underscores the important role of metallicity in the thermal transport properties of aqueous interfaces.

**Figure 7:**
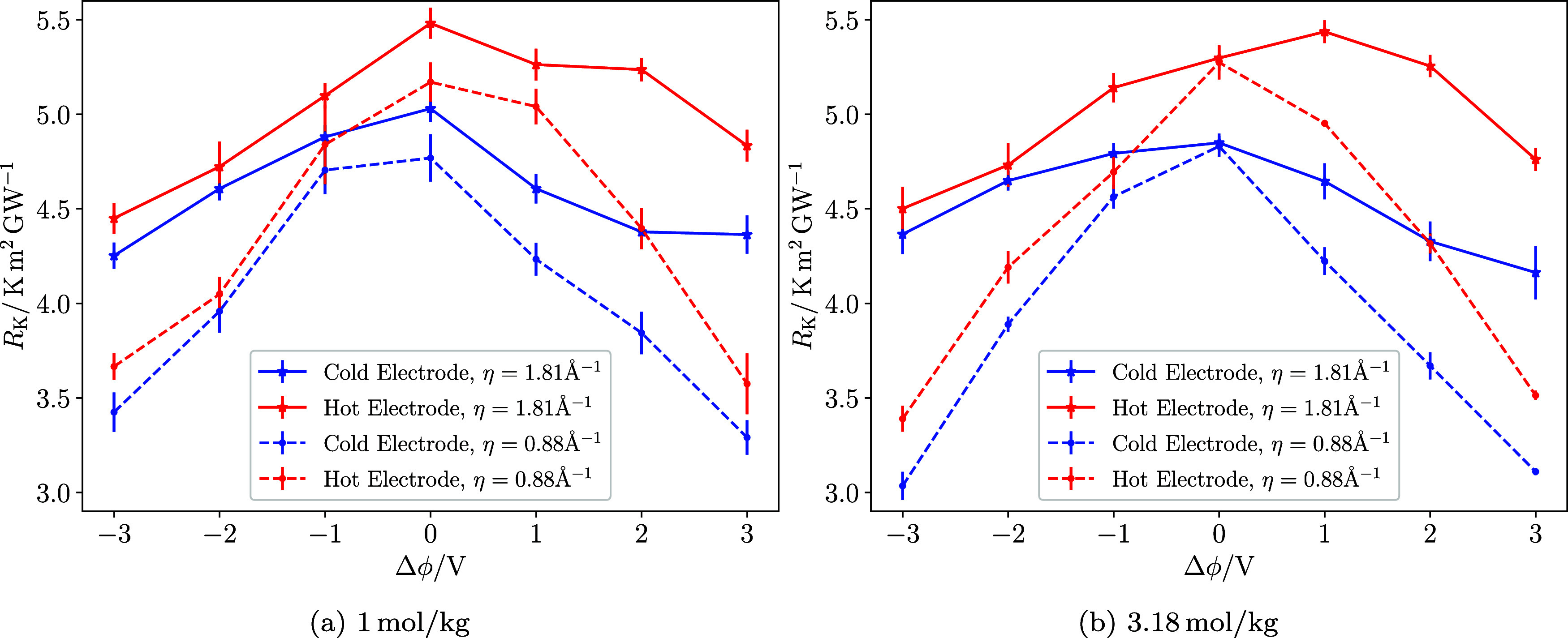
KR of NaCl systems confined between electrodes at 250 and 350 K as a function of the electrode potential difference. (a) 1 mol/kg NaCl system with LEM (solid line, η = 1.81 Å^–1^) and HEM (dashed line, η = 0.88 Å^–1^). (b) 3.18 mol/kg NaCl system with LEM (solid line, η = 1.81 Å^–1^) and HEM (dashed line, η = 0.88 Å^–1^). The blue and red lines represent the Kapitza resistance for the cold and hot electrodes, respectively. The potential difference is set such that Δϕ = ϕ_hot_ – ϕ_cold_. As in Figure 4, the use of Δϕ highlights the response to the imposed bias, whereas the corresponding comparison at equal surface charge density is shown in Figure 9.

When a potential of 3 V is applied, the electrodes exhibit surface charges of 10.8 μC/cm^2^ and 19.6 μC/cm^2^ for the LEM and HEM surfaces, respectively (see Supporting Tables S4a,b). These charges are significantly higher than those observed in the pure water system, particularly for the HEM electrodes. As mentioned in the previous section, an increased surface charge leads to a stronger interfacial molecular orientation of water, which enhances interfacial thermal transport, an effect also observed in electrolyte solutions (see Supporting Figure S17 for the orientation histograms).

We conducted additional simulations of NaCl solutions at a concentration of 3.18 mol/kg to analyze further the impact of salt concentration on the KR. Supporting Figure S11 shows the density profiles for the LEM 3.18 mol/kg system, for various applied potential differences. At a high concentration of 3.18 mol/kg, the KR of the cold electrode is again lower than that of the hot electrode (see Figure 7b). At 0 V, the KR values are similar to those calculated for the 1 mol/kg case (see Figure 7 left and right panels). The reduction in KR for LEM is 12–17% at 3 V, marginally lower than for the 1 mol/kg system.

The metallicity has a significant impact on how the KR varies with voltage in the 3.18 mol/kg system. As observed in water, the KR–voltage curves become more symmetrical as metallicity increases, reaching a maximum KR at 0 V. This behavior contrasts with that seen at LEM, where the maximum KR is achieved at the hot electrode at 1 V. With HEM surfaces, the reduction in KR at the cold electrode is much more drastic, showing a decrease of 33% and 36% at 3 V and −3 V, respectively. This reduction is greater than what was reported for the 1 mol/kg solution, highlighting a dependence of KR on salt concentration when the surfaces are highly metallic.

The reduction in KR is associated with several changes in interfacial properties, particularly the surface charge of the electrode, the orientation of water, and the distribution of ions. In the HEM scenario at 3 V, a significant peak in the Na^+^ density profile forms near the negative electrode, within 2 Å of the surface—closer than the oxygens of the first water layer (see Figure 8c). This inner peak appears at voltages greater than 2 V for both concentrations; however, it is only observed in HEM systems. At 2 V, the relative height of the inner peak is comparable for both concentrations. At 3 V, the peak height in the 1 mol/kg system remains unchanged. In contrast, the height of the inner peak in the 3.18 mol/kg system experiences a dramatic increase between 0 and 3 V—rising by approximately a factor of 5 (see Figure 8c).

**Figure 8:**
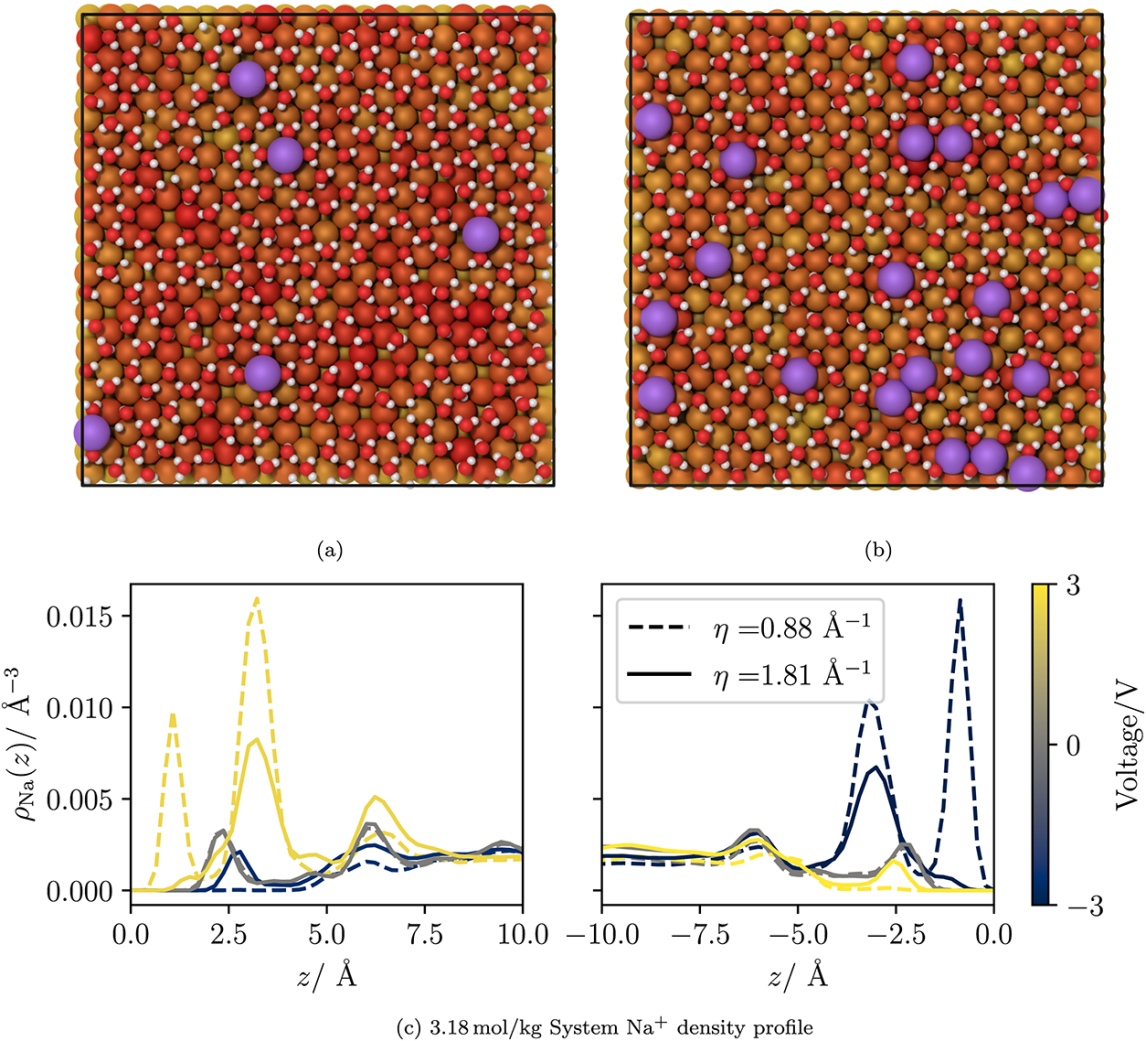
Snapshots depict the solution layer in contact with the electrode at a concentration of 3.18 mol/kg at 3 V with (a) LEM (η = 1.81 Å^–1^) and (b) HEM (η = 0.88 Å^–1^) electrodes. The purple spheres represent sodium ions. As with Figure 1, oxygen atoms are represented by red spheres, hydrogen atoms by white and metal atoms by orange spheres when charge neutral and dark red spheres for more negatively charged atoms. (c) Sodium density profiles for low and high metallicities at −3 V, 0 and 3 V.

The density peak closest to the electrode surface is due to the sodium ions adsorbing to the electrode surface differently. Rather than adsorbing on top of the metal atoms, as is the case of the low metallicity system, the ions occupy a position in the recess between four neighboring metal atoms. The alternative adsorption site allows the ions to approach much closer to the interface than would otherwise be possible. Additionally, the solvation of ions at this adsorption site differs. Water molecules surround the ion, forming a pattern resembling an “×”, with the cations positioned in the center (see Figure 8b). In contrast, in LEM electrodes, the ions are located on top of the metal atoms, while they are also surrounded by four neighboring water molecules forming a “+” pattern (Figure 8a). The distinct adsorption modes can be observed explicitly in panels (a) and (b) in Figure 8.

In their study of 1 mol/kg NaCl at 0 V with varying metallicities, Serva et al.^39^ refer to two adsorption modes as “Adlayer” (“+” shaped) and “Inner Sphere” (“×” shaped) adsorption. They observe the Inner Sphere adsorption only in small quantities at very high metallicities (η = 0.60 Å^–1^). Our results show this adsorption mode at a lower metallicity (η = 0.88 Å^–1^) when a strong voltage is applied. Additionally, a peak corresponding to the “Inner Sphere” adsorption is present in the 1 mol/kg system, but it is considerably weaker than in the 3.18 mol/kg system. The Inner Sphere adsorption mode was not observed when we set the Gaussian parameter to η = 1.81 Å^–1^ for either concentration of salt. Our simulations indicate that the solvation of ions interacting with highly metallic surfaces, along with the polarization charge induced on these surfaces, can overcome the direct repulsion between cations. This interaction leads to the formation of cation dimers and trimers at the electrode surface (see Figure 8b).

A preference for sodium ions to adsorb onto the hollow site has been observed in the DFT calculations conducted by Geada et al.,^75^ although this was studied at the Au(111) interface in a vacuum. This observation lends credibility to this mode of adsorption. The *ab initio* molecular dynamics study by Andersson et al.^51^ does not report this adsorption phenomenon. This difference may stem from the short time scales used in those simulations. In our own simulations, we do not find a significant population of this adsorption mode during the initial 1 ns run. This indicates that adsorption at this site is an activated process, likely driven by changes in ion solvation prior to ion attachment to the electrode surface.

The smallest thermal resistance we measured for our 3.18 mol/kg concentration at −3 V was 3.0 m^2^ K/GW. This value is significantly lower than the KR reported for carbon electrodes in contact with RTILs at similar voltages, which is around 18 m^2^K/GW.^35^ Additionally, our KR at the 3.18 mol/kg concentration using HEM electrodes is lower than the theoretical limit predicted for graphene-RTIL interfaces.^76^ Higher resistances, ranging from 16 m^2^K/GW to 52 m^2^K/GW, have been reported for water confined between graphene layers, particularly when using nonpolarizable surfaces.^72^ Under our simulation conditions, metallic electrodes exhibit lower interfacial thermal resistance than simulated carbon-based interfaces reported in the literature.

Higher or similar KR have been reported in studies involving confined water subjected to a constant electrostatic field or constant surface charge.^29,33,37^ However, the simulation conditions used in these studies differ significantly from those considered here. The CPM simulations involve a fluctuating electric field and the screening effects of the field generated by the electrodes. Moreover, the surface charges required to induce a significant reduction in the KR in constant charge calculations are often larger than those achieved in our CPM approach. For HEM electrodes, we obtained surface charges of ∼23 μC/cm^2^ at 3 V (see Table S5b in the SI for all voltages). These surface charges correspond to differential capacitances ranging from 10 μF/cm^2^ to 30 μF/cm^2^ for the 3.18 mol/kg system, significantly greater than the capacitance for the 1 mol/kg and pure water systems (10 μF/cm^2^ to 20 μF/cm^2^). These results underscore the importance of considering electrode polarization and charging effects to account for the metallicity of the electrodes.

One important aspect that still needs to be addressed is the microscopic mechanism behind the reduction of KR when electrodes are polarized and ions are present. Our analysis indicates that applying a higher potential leads to the accumulation of ions on the electrodes. These adsorbed ions create stronger charges on the electrodes, which enhance the interaction between the electrode and the ion and water molecules. The increased surface charges result in a stronger orientation of water molecules, thereby improving thermal transport. Therefore, the reduction in KR, which improves interfacial thermal transport, is associated with an increase in electrostatic interactions between the electrode surface and the solution. This effect can be achieved either by increasing the voltage or by adding salt, as both factors work together to enhance the polarization of the electrodes.

This notion is summarized in Figure 9, which illustrates the relationship between KR and the electrode surface charge density for all the systems examined in this study, including both hot and cold electrodes. The most notable aspect of this plot is that all systems can be represented by a single master curve, linking KR to the electrode charge density for the various systems investigated in this work. The plot also shows that the electrostatic potential offset observed at the maximum KR for LEM electrodes (see e.g., Figures 4 and 7) corresponds to zero electrode charge, helping to explain this behavior.

**Figure 9:**
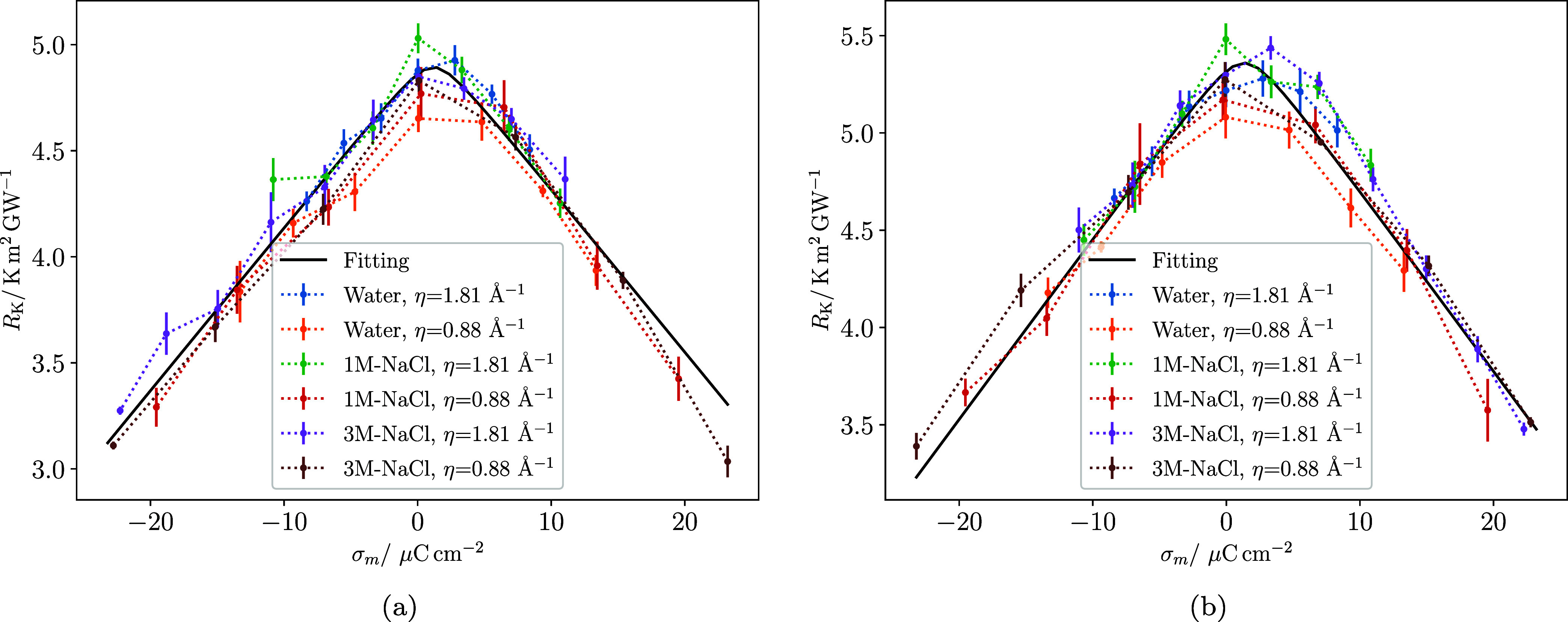
KR of all systems investigated as a function of the electrode surface charge. (a) Cold electrode interface. (b) Hot electrode interface. A linear fitting (black line in panel (a)) to the range >5 μC/cm^2^ gives 0.097 (m^2^K/GW)/(μC/cm^2^). We have performed a weighted fit of our data to the function, $R_{K}\left(\sigma_{m}\right)=R_{K,0}-\alpha \sqrt{{\left(\sigma_{m}-\sigma_{0}\right)}^{2}+w^{2}}+\alpha w$ . In the limit of σ – σ_0_ ≫ *w* this function becomes approximately linear, *R*_*K*_(σ_*m*_) = *R*_*K*,0_ – α­(σ_*m*_ – σ_0_) + α*w*. The fitting parameters are as follows: (a) *R*_*K*,0_ = 4.894 ± 0.056 K m^2^ GW^–1^, σ_0_ = 1.180 ± 0.235 μC cm^–2^, α = 0.0770 ± 0.0029 K m^2^ GW^–1^/(μC cm^–2^), *w* = 1.417 ± 1.274 μC cm^–2^; (b) *R*_*K*,0_ = 5.360 ± 0.077 K m^2^ GW^–1^, σ_0_ = 1.331 ± 0.166 μC cm^–2^, α = 0.0930 ± 0.0032 K m^2^ GW^–1^/(μC cm^–2^), *w* = 1.706 ± 1.348 μC cm^–2^.

The variation of the KR with surface charge could have a significant impact on heat transport at the nanoscale. To make this idea concrete, the Kapitza length, *L*_*K*_ = *R*_*K*_ *k*, provides a simple estimate of the thickness of fluid (with thermal conductivity *k*) that has an equivalent resistance to a single interface. Using our unbiased water–metal values *R*_*K*_ = 5 × 10^–9^ K m^2^/W and the water thermal conductivity *k*_H_2_O_ ∼ 0.6 W/(K m), one interface gives *L*_*K*_ ∼ 3 nm, and two interfaces in series (as in our system) ∼6 nm. Under strong polarization/metallicity, *R*_*K*_ drops to ∼3 m^2^K/GW, yielding *L*_*K*_ ∼ 1.8 nm per interface. In pores or gaps of 2–10 nm, this makes interfacial resistance comparable to the fluid’s internal resistance across the gap, so tuning *R*_*K*_ can meaningfully alter local heat flow. By contrast, for macroscale thicknesses, *L* ≫ *L*_*K*_, interfacial effects become negligible.

## Conclusions

We have demonstrated through constant potential nonequilibrium molecular dynamics simulations that the thermal transport at metal-water interfaces is influenced by the electrode metallicity, applied potential, and electrolyte composition.

Our results indicate that increasing the metallicity of the electrodes significantly enhances heat transfer across the electrode–solution interface. The decrease in interfacial Kapitza resistance emerges from greater differential capacitance, leading to stronger electrode polarization. This polarization leads to a more ordered arrangement of water molecules near the surface, ultimately facilitating more efficient energy exchange.

Applying external potentials reduces the Kapitza resistance, irrespective of the electrode’s polarity. This effect is mainly due to the voltage-induced restructuring of the interfacial water layer. We observe a similar enhancement in thermal transport for both positive and negative electrodes.

The introduction of salt adds complexity and works synergistically with voltage, strengthening the electric double layer and enhancing the orientation of water molecules, thereby improving thermal transport. At higher salt concentrations, these effects become even more pronounced. Our data show that *R*_*K*_ is primarily governed by the electrode surface charge density σ, as indicated by the collapse of all results for all fluids, metallicities, and voltages onto a single curve. Regarding synergy, metallicity and salt concentration influence *R*_*K*_ through their joint impact on the observed σ at a given bias, by raising the differential capacitance and compacting the double layer. Therefore, our results suggest that varying the composition of the electrolyte and the metallic properties of the electrodes is an effective method for tuning interfacial heat flow.

The analysis of water dipole orientation distributions reveals that LEM electrodes exhibit a mixture of two predominant water configurations, whereas highly metallic surfaces enforce a single, strongly aligned orientation. This increased ordering of water molecules has an associated reduction in thermal resistance, underscoring the importance of the orientation of interfacial water molecules in thermal transport. Our examinations of charging dynamics and capacitance demonstrate that electrode polarization and interfacial structuring are closely interconnected. In contrast, the analyses of vibrational density of states suggest that vibrational coupling is relatively unaffected by the applied voltage within the studied range.

The lowest KR observed in our study, ≈ 3.0 m^2^K/GW for a 3.18 mol/kg NaCl solution at −3 V, is substantially lower than those reported using classical force fields, for carbon electrodes in contact with room-temperature ionic liquids (≈ 18 m^2^K/GW). It also falls below the theoretical limit predicted for graphene–RTIL interfaces.^76^ For RTIL-carbon interfaces, an increase in voltage of 3 V results in a reduction of ∼20% in the Kapitza resistance of these interfaces,^35^ which is lower than the ∼35% reduction observed in the aqueous interfaces investigated here, at HEM and high salt concentration. Previous studies on water confined between graphene layers reported much higher resistances (13–52 m^2^K/GW), particularly for nonpolarizable surfaces.^72,77^ These comparisons with previous simulations performed with similar classical force fields suggest that highly metallic electrodes combined with aqueous electrolytes provide significantly better conditions for interfacial heat transfer than less polarizable carbon-based systems.

We have demonstrated that the general dependence of the Kapitza resistance of the systems investigated here on the applied voltage follows a master curve. The Kapitza resistance changes approximately linearly with the electrode surface charge. Based on the analysis of all the systems investigated in this work, we estimate a reduction of the Kapitza resistance of 0.077–0.093 K m^2^/GW per μC/cm^2^ of surface charge. Hence, electrodes with a higher differential capacitance provide a better choice to enhance interfacial thermal transport by varying the electrostatic potential. Our predictions suggest targeted experiments under bias to measure interfacial thermal resistance vs electrostatic potential. To our knowledge, direct measurements of KR under applied bias at metal–aqueous interfaces have not yet been reported, although the KR has been measured in other contexts using e.g., thermoreflectance techniques.^11,16^

We note that our constant-potential simulations use nuclei-centered Gaussian charges to enforce equipotential electrodes and enable large-scale, nonequilibrium simulations with controlled bias and steady heat flux. However, CPM does not include explicit electronic structure, so quantum effects such as electron spillover into interfacial water, known to increase Helmholtz capacitance, are not included explicitly. Adjusting the Gaussian width η tunes “metallicity” and can improve agreement with capacitance and promote specific cation adsorption. However, absolute KR values and detailed adsorption motifs may vary with electronic spillover and water flexibility. In particular, rigid vs flexible water contributions in librational spectra and fine structure of the first layer may shift orientation histograms slightly. We, however, expect that the qualitative charge-orientation-KR correlation will be preserved. In particular, our analysis of the metallicity parameter, η, indicates a practical route to raise differential capacitance (choice of metal, surface structure, or electrolyte composition) and modify the Kapitza resistance accordingly. Future extensions of this work might involve exploring CPM variants with semiclassical electron-spillover corrections and/or DFT-trained Machine Learning Force Fields, aiming to combine large-scale nonequilibrium simulations with improved electronic realism under bias and heat flux. Furthermore, additional studies are needed to test the KR-surface charge relationship demonstrated in our work, targeting other electrolytes, solvents and electrode materials.

Our findings indicate that interfacial thermal transport at electrode–water interfaces is not a fixed property. Instead, it can be influenced by selecting or engineering the electrode material and surface. This insight opens opportunities for developing electrotunable thermal management strategies in electrochemical systems, energy storage devices, and nanoscale heat transfer applications. Under realistic operating biases, selecting electrodes with higher effective metallicity can lower the interfacial KR, reducing temperature discontinuities and enhancing passive heat extraction at metal–aqueous interfaces.

## Supplementary Material



## Notes

Comparison between the potential profiles of the unpatched LAMMPS version and the patched version, using the CG algorithm and using the matrix inversion algorithm; cumulative energy added or removed from the cold and hot thermostat regions for a representative simulation of pure water; fitting parameters of water charging dynamics for the data presented in Figure S4; electrostatic potential profiles of water at different applied voltages; distributions of the HOH angle bisector orientation in the first water layer of the 3.18 mol/kg NaCl system (PDF)
