# Supplementary material for: Electrotunable Kapitza Resistance at Electrode-Water Interfaces: The Importance of Electrode Metallicity
Source: J Phys Chem C Nanomater Interfaces. 2026 May 7;130(20):7086–99. doi: 10.1021/acs.jpcc.6c00351 (PMC13200241; doi:10.1021/acs.jpcc.6c00351)
Supplement: Supplementary file 1 [file jp6c00351_si_001.pdf]

Supporting Information: Electrotunable Kapitza Resistance at Electrode-Water Interfaces: The Importance of Electrode Metallicity

Aidan Chapman* and Fernando Bresme*

Department of Chemistry, Molecular Sciences Research Hub, Imperial College, W12 0BZ, London, United Kingdom

E-mail: aidan.chapman16@imperial.ac.uk; f.bresme@imperial.ac.uk

S1 Simulation Details

To model the interatomic interactions of the metallic electrodes we used the Lennard-Jones 12–6 parameters for gold by Heinz et al.[S1]. Water was modelled using the SPC/E force field.[S2] The molecules were held rigid using the SHAKE[S3] constraint algorithm on the bonds and angles, with a tolerance of 10^{-4} and a maximum of 20 iterations per timestep. In systems with salt added (1M NaCl, 3M NaCl) we utilised the force field by Dang[S4] for the ion interactions. Cross interactions between these three components used the geometric mixing rules for the Lennard-Jones parameters ($\epsilon_{ij} = \sqrt{\epsilon_i \epsilon_j}$, $\sigma_{ij} = \sqrt{\sigma_i \sigma_j}$). This set of force fields has been used by several other constant potential prior studies. [S5,S6]

The electrostatic interactions were computed using the PPPM[S7,S8] method with a tolerance of 1×10^{-7}.

Fully periodic boundaries were implemented, and a region of vacuum was included to prevent interactions with the images of the two electrodes. However, the metallic surfaces remain electrically connected and enforced to have equal and opposite total charges, ensuring that the overall system maintains charge neutrality. The vacuum is unnecessary for preventing dipole interactions between the system's images, thanks to the finite field method.[S9] Nevertheless, it does serve to prevent direct dispersion interactions between the two electrodes across the periodic images.

Each system was run for 5 or 10 independent repeats, each lasting 1.0 ns. All values reported, unless otherwise stated, are averages over these repeats, with error bars reported as the standard errors. All the simulations were performed with a timestep of 2.0 fs.

The simulations were performed using a modified version of the LAMMPS simulation software[S10] and makes use of the ELECTRODE[S6] package within LAMMPS. The modifications are detailed in the Subsection S2.1.

S2 Details on Constant Potential Method

In the constant potential method the charges of the N electrode atoms are dynamically readjusted so that the potential each atom experiences is equal to a target value, Φ. In this work, the potential difference between two electrodes is specified as $\Delta\Phi$ and each electrode is set to $\pm\frac{\Delta\Phi}{2}$.

Under standard periodic boundary conditions the electrostatic contributions of the electrode atoms to the potential energy can be written in the form,[S11,S12]

$$V_{\text{elec}}(\mathbf{r}^M, \mathbf{r}^N, \mathbf{q}) = \frac{1}{2}\mathbf{q}^T \mathbf{A}(\mathbf{r}^N)\mathbf{q} - \mathbf{q}^T \mathbf{b}(\mathbf{r}^M), \tag{1}$$

where $\mathbf{r}^N$ and $\mathbf{r}^M$ are the positions of the electrode and electrolyte atoms, respectively, the matrix $\mathbf{A}$ has the shape $(N,\ N)$ and represents potential contributions due to the electrode-electrode atom Coulombic interactions and depends only on the electrode atom positions. The vector $\mathbf{b}$ is the per-atom potential due to the electrode-electrolyte interactions, which has the length N. The explicit forms of $\mathbf{A}$ and $\mathbf{b}$ in the PPPM method variant of the constant potential method — which has been used in this work — are given ref.[S8]

Periodic boundaries are at odds with the non-periodic nature of a typical two electrode simulation set up. One approach that rectifies this issue is the finite-field variation of the constant potential method.[S9] This method treats the two electrodes as a single conductor with a uniform field applied across it,

$$\mathbf{E}_{\text{ff}} = -\frac{\Delta\Phi}{L_z}\hat{\mathbf{z}}, \tag{2}$$

where $\hat{\mathbf{z}}$ is the unit vector between the two electrodes and L_z is the simulation box length along that direction. This field compensates for the dipole moment of the simulation box.

In the original finite field work, the simulations were performed with a single electrode in the centre of the simulation box.[S9]

Because the polarization of a simulation box is a multivalued property — and only changes affect the dynamics — we can shift the electrodes around to calculate the polarization of different images of the simulation box. To match the polarization of the electrode-centred setup of Dufils et al.[S9], the electrode atoms can be shifted around for the polarization calculation. This was achieved in the original LAMMPS implementation[S6] by shifting the lower electrode by L_z. In our modified version we shift the atoms of both the lower and upper electrodes by $-\frac{Lz}{2}$ and $\frac{L_z}{2}$, respectively.

Using the shifted coordinates the electrostatic potential energy of the system can be written as,[S6,S9],

$$V_{\text{elec,ff}}(\mathbf{r}^M, \mathbf{r}^N, \mathbf{q}) = V_{\text{elec}}(\mathbf{r}^M, \mathbf{r}^N, \mathbf{q}) - \sum_{i=1}^{N} q_i z'_j \cdot \mathbf{E}_{\text{ff}} \tag{3}$$

$$= \frac{1}{2}\mathbf{q}^T \mathbf{A}(\mathbf{r}^N)\mathbf{q} - \mathbf{q}^T \mathbf{b}(\mathbf{r}^M) - \sum_{i=1}^{N} q_i z'_j \cdot \mathbf{E}_{\text{ff}}, \tag{4}$$

where z'_j is the z coordinate of the electrode atom j transformed such that the electrodes are centered in the simulation box.

The charges on the electrode atoms can be determined by minimizing the quadratic equation, $V_{\text{elec,ff}} - \sum_{i=1}^{N} q_i \Phi_i$ with respect to the electrode charges, q_i, for target atom potentials Φ_i. Equivalently, the electrostatic potential on atom i, $\frac{\partial V_{\text{elec,ff}}}{\partial q_i}$ can be equated to its target potential and the resulting system of N linear equations can be solved using linear algebra techniques.

S2.1 Modifications Required to LAMMPS

One issue with using dynamic electrodes is that the default method for calculating the capacitance matrix (the inverse of $\mathbf{A}$) in LAMMPS,[S6] is too computationally expensive to be calculated at every single timestep. The use of a frozen electrode enables the matrix $\mathbf{A}$ and its inverse to be calculated just once at the start of the simulation and reused on

subsequent timesteps. This cannot be done when the electrode atoms move — the matrix **A** depends on those electrode positions and must be recalculated at every step. As such, it can not be used for practical calculations with dynamic electrodes, without modification to the source code. However, the Conjugate Gradient (CG) method for matrix inversion is also implemented in the ELECTRODE[S6] package and does support dynamic electrodes.

The CG method iteratively approximates solutions to the system of equations, down to some tolerance. Generally, the CG method seeks to numerically solve linear equations of the form,[S13]

$$\mathbf{M}\mathbf{x} - \mathbf{v} = \mathbf{0} \tag{5}$$

where $\mathbf{M}$ is a square matrix of size $n \times n$ and $\mathbf{x}$ and $\mathbf{v}$ are vectors with n elements. The implementation of this algorithm is in LAMMPS based on the pseudocode provided in the thesis of Gingrich[S14]. This version adds in constraints to ensure charge symmetry of the two electrodes.

Combining the CG method with the finite-field version of CPM required modifications to LAMMPS. Without these, LAMMPS silently ignores the `ffield` option when running with the CG algorithm for matrix inversion, and the namesake finite field was not included in the calculation of the potential. This resulted in the calculated electrode potentials being drastically different from the target values.

To correct this, the finite field was re-added, by using the CG solver on,

$$\mathbf{q} = \mathbf{A}^{-1}\left(\mathbf{b} - z_i'\frac{\Delta\phi}{L_z}\right), \tag{6}$$

where z_i' is electrode atom z coordinate transformed into the cell centred geometry, in our

case this was achieved by shifting both the left and right electrodes,

$$z_i' = \begin{cases} z_i + \frac{L_z}{2}, & \text{if } i \in \mathcal{L} \\ z_i - \frac{L_z}{2}, & \text{if } i \in \mathcal{R} \end{cases} \tag{7}$$

where $\mathcal{L}$ and $\mathcal{R}$ are the sets of atoms in the left and right electrodes, respectively. The centred electrode geometry ensures the potential energy due to the finite field (the $\mathbf{E} \cdot \mathbf{P}$ term) is continuous across the PBCs. Note that this method of centring the electrodes is different to how it was implemented for the matrix inversion algorithm;[S6] in that case, only the atoms in the lower electrode are shifted, by a single box length.

Figure S1 shows the potential profiles for various solver methods, with both patched and unpatched versions of LAMMPS. The system is the aqueous NaCl and gold finite field example that is included with LAMMPS, under a 2.0 V potential difference. The potential profile is calculated as discussed in Section S6.3 in this SI. The modified versions of LAMMPS used here (red line) has the correct potential profile when using the CG solver that matches that of the matrix inversion method (teal line) — the unaltered version of LAMMPS using the CG solver (blue line) does not.

All our simulations were thus run using a modified version of LAMMPS. The water and 1 mol/kg NaCl simulations used a version of LAMMPS forked circa June 2023 of LAMMPS.[S15] With the patch applied, the git ref was 459d0b30b3dc. The 3.18 mol/kg simulations ran with the patch applied to the Stable August 2023 version of LAMMPS. The patch is available in a GitHub repository.[S15]

This patch also includes another fix. The ELECTRODE package has an optimization that requires all the atoms in an electrode to be only present in that electrode.[S6] To ensure that no electrode atom types are present elsewhere in the system, the ELECTRODE package makes the assumption that at least one atom of every type is present. However, when this is not the case it erroneously reports that a non-electrode atom is present outside the electrode group and refuses to run the simulation.

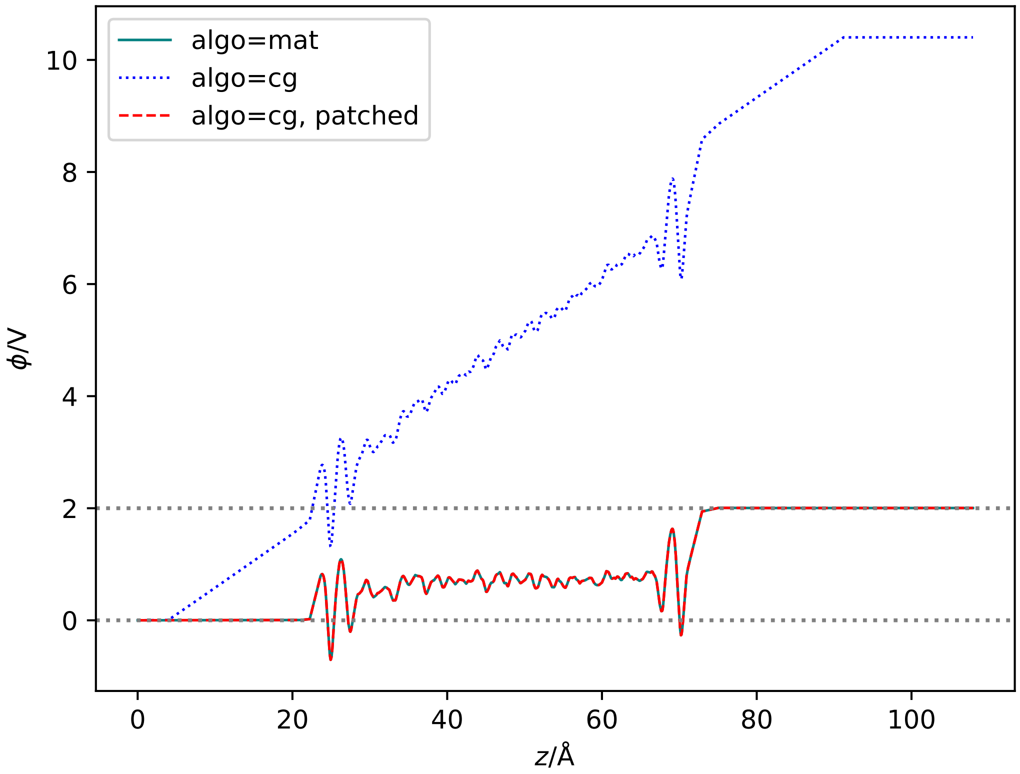

Figure S1: Comparison between the potential profiles of the unpatched LAMMPS version and the patched version, using the CG algorithm and using the matrix inversion algorithm. The unpatched version is a development version from June 2023.

S3 Energy exchanged at the hot and cold thermostats

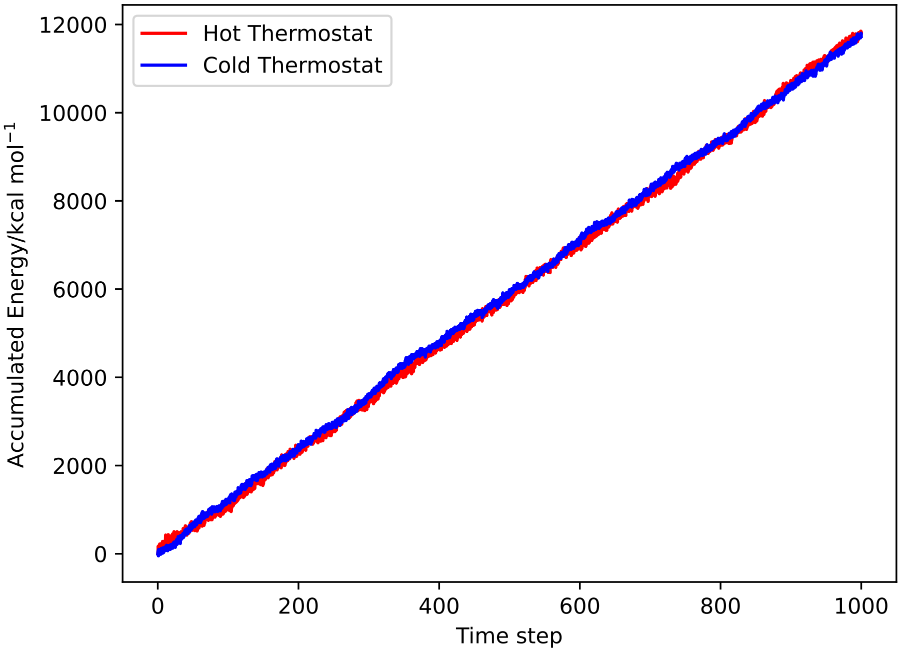

Figure S2: Cumulative energy added or removed from the cold and hot thermostat regions for a representative simulation of pure water. The thermostats are set to 350 and 250 K. The energy exchanged at the cold or hot thermostat is > 0 or < 0. To facilitate comparison, the results for the hot thermostat are shown in absolute value.

S4 Calculation of the Kapitza resistance

The central quantity discussed in the main paper is the Kapitza resistance. This is given by eq. (1) from the main text,

$$R_{\mathrm{K}} = \frac{\Delta T}{J_q},$$

where ΔT is the interfacial temperature "jump" and J_q is the heat flux normal to the interface.

$$J_q = \frac{1}{A}\dot{Q},$$

where A is the cross-sectional surface area and $\dot{Q}$ is the heat rate, which is determined from the a linear fitting of the average energy cumulative energy exchange at the hot and cold thermostats (see Figure S2). To calculate the temperature profiles, we use the equipartition equation. For water we employed the oxygen and hydrogen degrees of freedom reported in reference.[S16]

For the purposes of determining the temperature jumps, ΔT, the location of the interface was defined as the left(right) most bin with a non-zero density of electrode atoms for the left(right) interface. This is approximately the location of where the electrode density drops to zero after the peak corresponding to the outermost electrode layer (see vertical dashed grey lines in Figure S3).

The raw temperature profiles, which are split by atom type, output from LAMMPS feature unphysical drops in temperature. This occurs when the number of atoms in the interfacial region drops to zero, and the averaged temperature reported becomes incorrectly low (blue dashed lines in Figure S3). A similar issue occurs between the layers of the electrode atoms (gold dashed lines in Figure S3).

To determine the temperature on the electrolyte side of the interface, the electrolyte

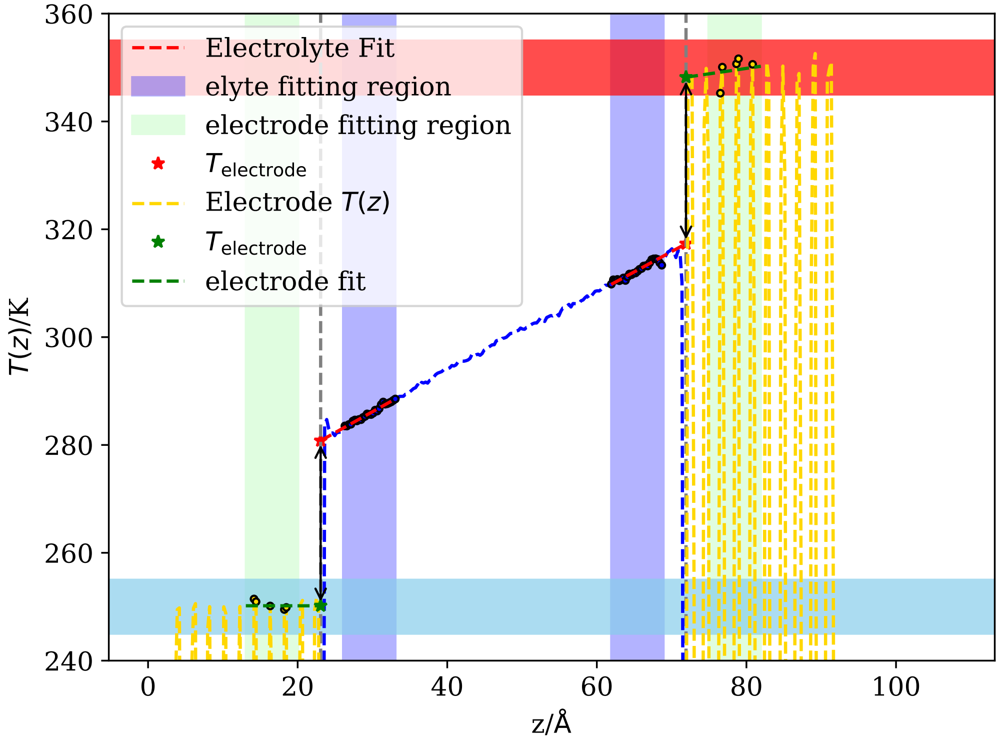

Figure S3: Determining the electrolyte-electrode temperature discontinuity, using a water system with $\eta = 1.81\,\text{Å}^{-1}$ and $(T_{\text{low}}, T_{\text{high}}) = (250\,\text{K}, 350\,\text{K})$ as an example. The dashed blue and yellow lines represent the electrolyte (elyte) and electrode temperature profiles, respectively. The blue (yellow) points are those used in the fittings for determining the temperature on the electrolyte (electrode) sides of the interface. The dashed grey vertical lines indicate the locations of the electrode-electrolyte interface. The vertical shadowed regions in blue and green, indicate where the temperature profiles were fitted for extrapolation to the interface. The horizontal spans are $\pm 5\,\text{K}$ from the electrode target temperature — only electrode temperatures within this range were used for fitting purposes. The dashed red and green lines are the fits for the electrolyte and electrode temperature profiles, respectively.

temperature profile was fitted linearly and extrapolated to the interface. The fitting was performed within the regions,

$$z_{\text{left}} + b < z < z_{\text{left}} + l,$$

and

$$z_{\text{right}} - l < z < z_{\text{right}} - b,$$

for the left and right interfaces, respectively. In these equations, z_{left} and z_{right} are the previously discussed interface locations, $b = 3\,\text{Å}$ is a buffer away from the interface, added to avoid the aforementioned unphysical drops in temperature due to sampling, and $l = 10\,\text{Å}$ is the maximum distance the temperature profile is assumed to be linear away from the electrode interface. The regions are illustrated by the blue spans in Figure S3. The linear fittings are weighted by the density of the bin and then used to extrapolate the temperature to the interface location.

For the electrode side of the interface, the temperature was linearly fit to the bins with temperatures within 5 K of the thermostat target temperature (see horizontal spans in Figure S3) This criterion is used once again to remove the unphysical temperature drops. Once again, the fitting to the temperature profile is over the region between 10 Å to 3 Å away from the interface (see green spans in Figure S3).

The interfacial temperature "jump", ΔT, was then calculated by taking the difference in the temperatures obtained by extrapolating to the interface from either side.

S5 Charging dynamics

The time-dependent electrode charge was fitted with the equation,

$$Q(t) = Q_{st}\left[1 - a\exp(-t/\tau)\right] \tag{8}$$

where Q_{st}, is the electrode charge in the stationary state, a is a fitting constant, and τ quantifies the charging time.

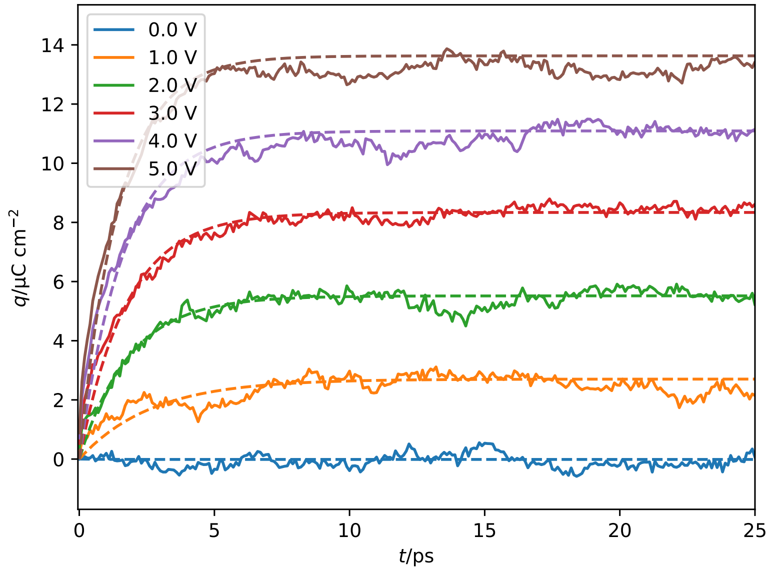

Figure S4: The charging dynamics of the hot electrode for several potentials for the pure water system with electrodes at 250 K–350 K and $\eta = 1.81$ Å^{-1}. Due to charge symmetry, the cold electrodes have equal and opposite charge. The dashed lines represent fittings to Equation (8) The parameters of these fittings are shown in Table S1.

Table S1: Fitting parameters of water charging dynamics for the data presented in Figure S4.

$\Delta\phi$/V	q_∞/µC/cm^2	τ/ps
0.000000	−0.0130	0.6475
1.000000	2.7033	2.6608
2.000000	5.5172	1.8476
3.000000	8.3360	1.7605
4.000000	11.0920	1.6629
5.000000	13.6298	1.4848

S6 Supporting data for the computations of pure water systems

S6.1 Summary of Simulation Results

Tables S2a and S2b summarise key results and simulation conditions for the water system, for the low- and high-metallicity electrodes, respectively.

Table S2: Results for the water system with low metallicity ($\eta = 1.81\,\text{Å}^{-1}$) electrodes. The columns T_l, T_r and $\Delta\phi$ are the target temperatures for the left and right electrodes, and the target potential difference, respectively. The subscripts l and r refer to the 'left" and "right" electrodes. The column p is the average zz (normal to the electrode surface) component of the pressure tensor. The column J_q is the heat flux resulting from the applied temperature difference, calculated as discussed in section S4. The columns $\delta\phi_\text{l}$ and $\delta\phi_\text{r}$ are differences in potential between the electrode surface and the second minimum in the water density. The columns $R_{\text{K,l}}$ and $R_{\text{K,r}}$ are Kapitza Resistances at the two interfaces. The numbers in parentheses indicate the uncertainty (standard error), e.g. -391(6) means -391±0.6.

T_l/K	T_r/K	$\Delta\phi$/V	p/atm	J_q/GW/m^2	$\delta\phi_\text{l}$/V	$\delta\phi_\text{r}$/V	σ_l/μC/cm^2	σ_r/μC/cm^2	$R_{\text{K,l}}$/Km^2/GW	$R_{\text{K,r}}$/Km^2/GW	ΔT_l/K	ΔT_r/K
250	350	-3.000	−391(6)	6.65(5)	−1.745(6)	0.771(5)	8.389(6)	−8.389(6)	4.50(7)	4.66(5)	29.9(5)	31.0(4)
250	350	-2.000	−349(5)	6.47(2)	−1.245(4)	0.468(6)	5.563(8)	−5.563(8)	4.77(5)	4.85(8)	30.8(3)	31.4(5)
250	350	-1.000	−329(7)	6.27(4)	−0.761(5)	0.101(5)	2.785(5)	−2.785(5)	4.93(7)	5.14(8)	30.9(4)	32.2(4)
250	350	0.000	−322(6)	6.21(5)	−0.313(5)	−0.311(8)	0.021(5)	−0.021(5)	4.88(6)	5.22(9)	30.3(3)	32.4(5)
250	350	1.000	−332(7)	6.34(4)	0.105(6)	−0.750(6)	−2.731(6)	2.731(6)	4.65(7)	5.28(9)	29.5(4)	33.5(6)
250	350	2.000	−357(5)	6.42(3)	0.479(5)	−1.239(7)	−5.522(8)	5.522(8)	4.54(6)	5.2(1)	29.1(5)	33.4(7)
250	350	3.000	−398(3)	6.59(5)	0.804(4)	−1.742(8)	−8.306(8)	8.306(8)	4.26(5)	5.01(9)	28.1(3)	33.0(5)

(a) $\eta = 1.81\,\text{Å}^{-1}$

T_l/K	T_r/K	$\Delta\phi$/V	p/atm	J_q/GW/m^2	$\delta\phi_\text{l}$/V	$\delta\phi_\text{r}$/V	σ_l/μC/cm^2	σ_r/μC/cm^2	$R_{\text{K,l}}$/Km^2/GW	$R_{\text{K,r}}$/Km^2/GW	ΔT_l/K	ΔT_r/K
250	350	-3.000	$-4.0(1) \times 10^2$	7.14(4)	−1.666(7)	0.401(3)	13.35(2)	−13.35(2)	3.94(7)	4.18(8)	28.1(4)	29.8(6)
250	350	-2.000	$-2.7(1) \times 10^2$	6.91(6)	−1.252(6)	0.217(8)	9.37(2)	−9.37(2)	4.31(3)	4.41(3)	29.8(3)	30.5(3)
250	350	-1.000	−193(6)	6.65(4)	−0.787(3)	−0.026(8)	4.81(2)	−4.81(2)	4.64(9)	4.85(8)	30.8(6)	32.2(5)
250	350	0.000	−181(8)	6.49(6)	−0.357(6)	−0.35(1)	0.05(1)	−0.05(1)	4.65(6)	5.1(1)	30.2(4)	33.0(5)
250	350	1.000	−203(5)	6.61(6)	−0.014(5)	−0.776(8)	−4.702(5)	4.702(5)	4.31(9)	5.01(9)	28.5(7)	33.1(5)
250	350	2.000	−271(7)	6.91(6)	0.226(8)	−1.254(4)	−9.32(2)	9.32(2)	4.16(7)	4.6(1)	28.7(5)	31.9(7)
250	350	3.000	−397(8)	7.25(6)	0.410(7)	−1.677(6)	−13.30(2)	13.30(2)	3.8(1)	4.3(1)	28(1)	31.1(7)

(b) $\eta = 0.88\,\text{Å}^{-1}$

S6.2 Density profiles

Figure S5 shows the density profiles of hydrogen and oxygen atoms at different voltages and metallicity parameter, η =1.81 Å.

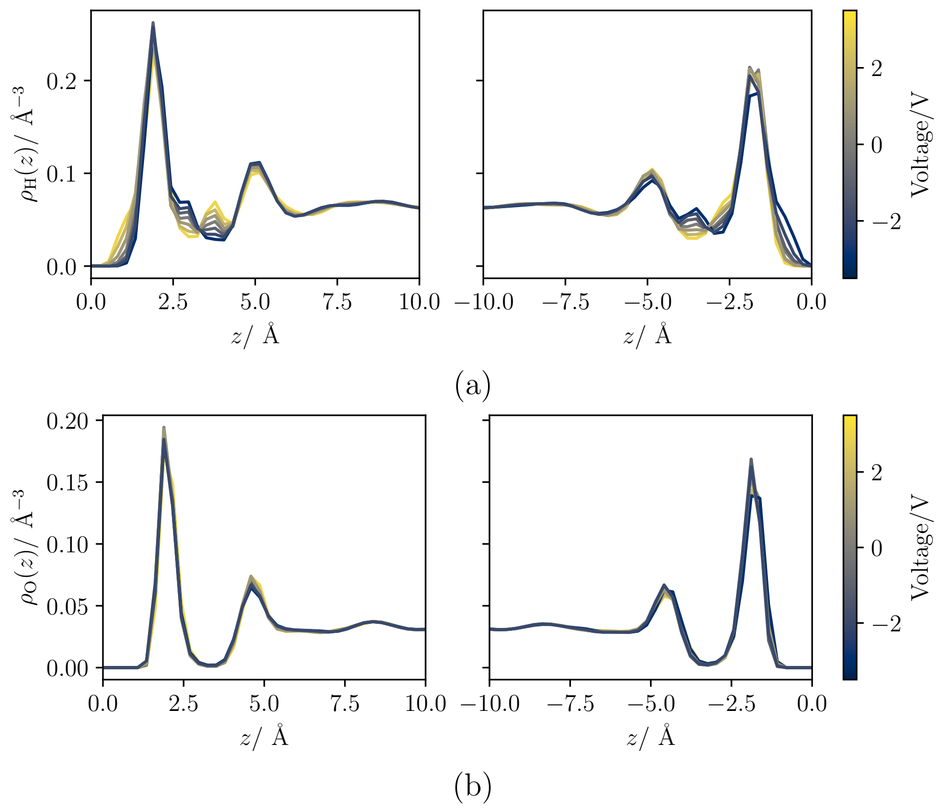

Figure S5: Hydrogen (a) and oxygen (b) number density profiles of the pure water system simulated with thermostats set at 250 and 350 K and at different voltages. The metallicity parameter was set to $\eta = 1.81\,\text{Å}^{-1}$. The left and right panels correspond to the density at the left and right electrodes, respectively. The x-axis indicates the distance from the electrode-water interface plane.

S6.3 Potential Profiles

Figure S6 shows the electrostatic potential profiles for pure water subjected to a thermal gradient. These were calculated by twice integrating the charge density along the z-axis,

$$\phi(z) = -\frac{1}{\varepsilon_0} \int_0^z \int_0^{z'} \rho(z'')\, \mathrm{d}z'\, \mathrm{d}z''\,. \tag{9}$$

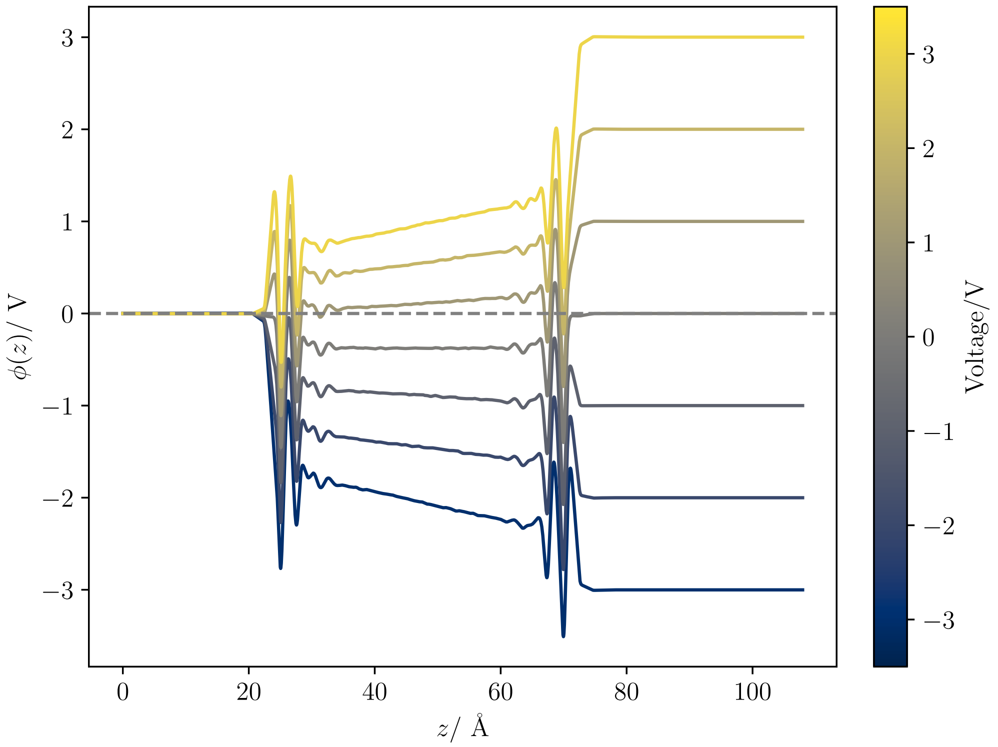

Figure S6: Electrostatic potential profiles of water at different applied voltages. The temperature of the electrodes are 250 and 350 K. The metallicity parameter was set to $\eta = 1.81\,\text{Å}^{-1}$.

S6.4 Water in plane ordering

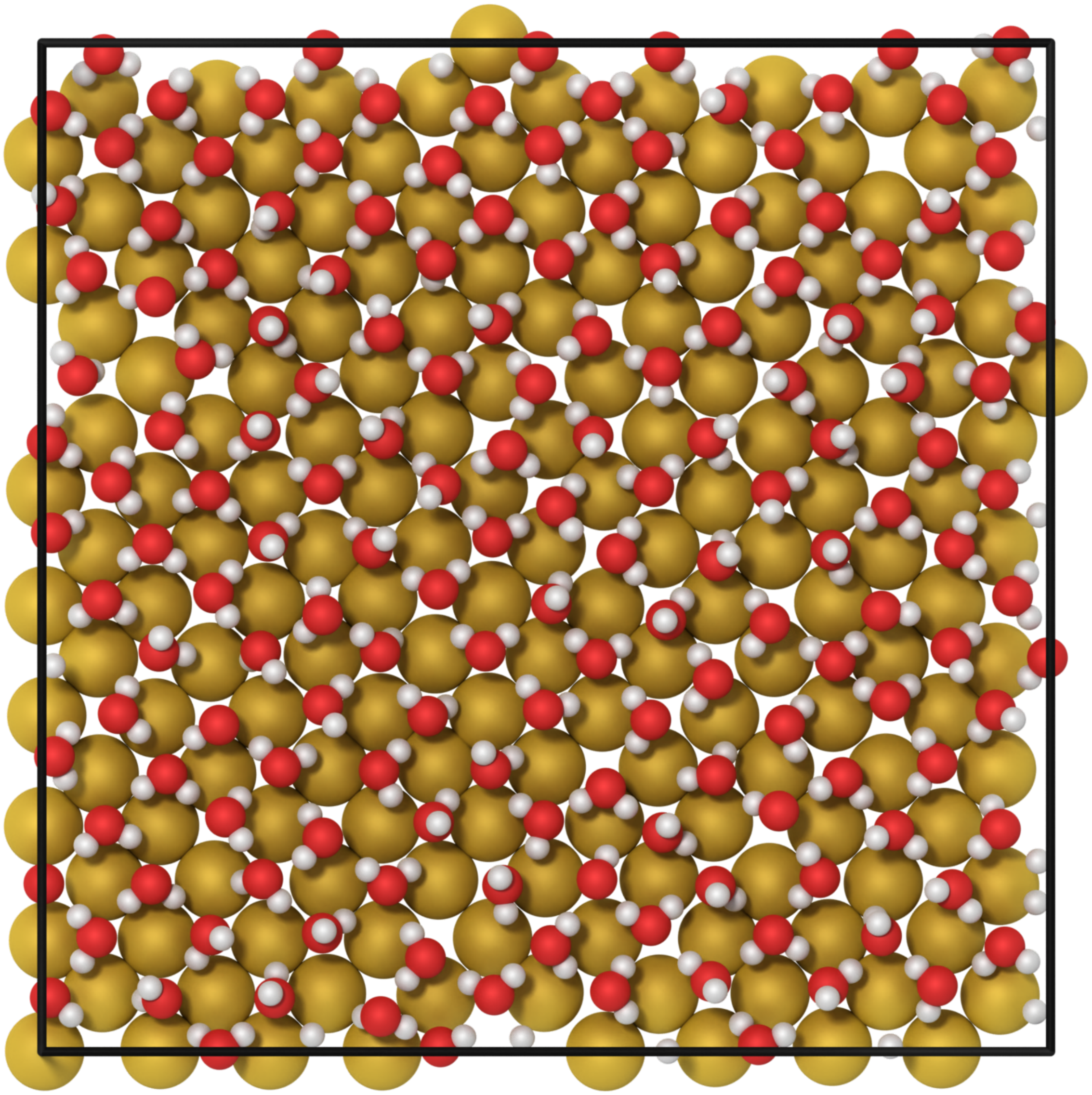

Figure S7: Snapshot of the hot interface of the water system with 0 V applied voltage and low metallicity electrode. Generated with OVITO Pro.[S17]

S7 Surface charge and capacitance

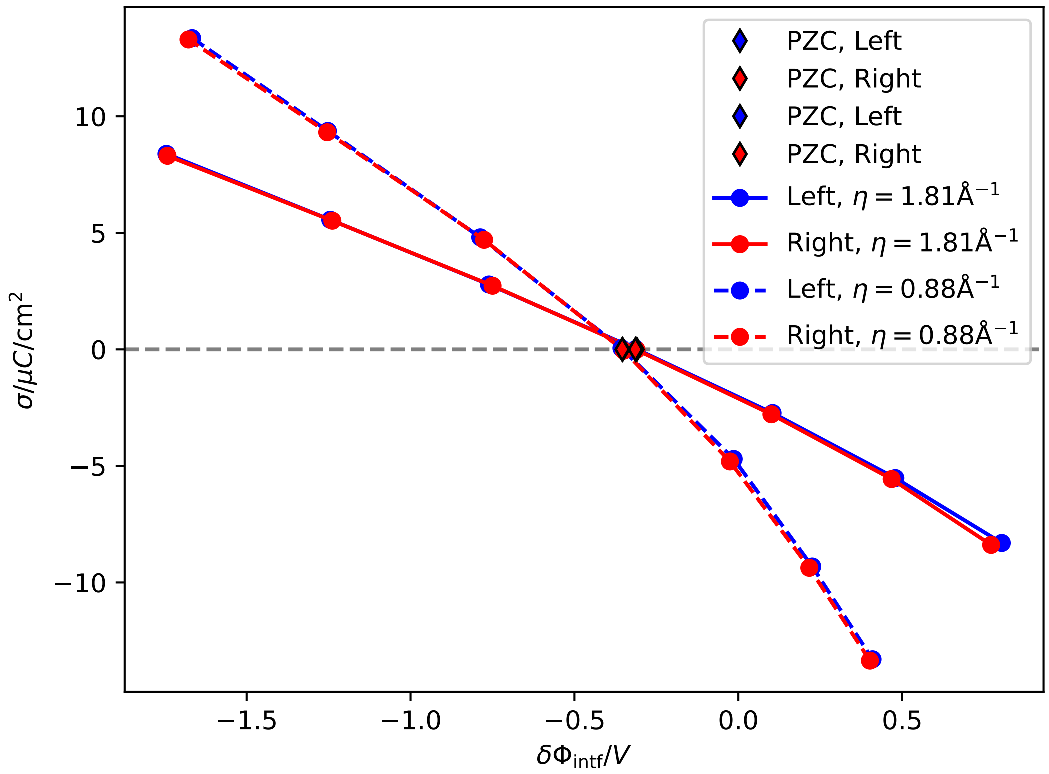

Figure S8: Illustration of the determination of the potential of zero charge (PZC) from surface charge densities and surface potentials in the pure water system.

Table S3: The potential of zero charge (PZC) and differential capacitances for all systems investigated in this work. Every system has the cold electrode set to $250\,\mathrm{K}$ and hot electrode to $350\,\mathrm{K}$. The surface potential at zero charge $\delta\phi_{\mathrm{PZC}}$ was determined from x-axis intersection of the electrode charge against surface potential plot (see Figure S8 for an example). The two capacitances reported were calculated from linear fits above and below the PZC to plots of electrode charge against surface potential relative to the PZC (see Figure S9). The suffixes $<\mathrm{PZC}$ and $>\mathrm{PZC}$ indicate fittings performed below and above the PZC, respectively. The values of the PZC were found using Newton's method on the (linearly interpolated) surface charge as a function of the surface potentials. The default tolerances for the `scipy.root_scalar` method were used. The numbers in parentheses indicate the uncertainty (standard error).

Electrolyte	$\eta/\text{Å}^{-1}$	Interface	$\delta\phi_{\mathrm{PZC}}/\mathrm{V}$	$C_{D,<\mathrm{PZC}}/\mu\mathrm{F/cm}^2$	$C_{D,>\mathrm{PZC}}/\mu\mathrm{F/cm}^2$
Water	0.88	Cold	-0.353	10.1(2)	17(1)
		Hot	-0.354	10.0(3)	18(1)
	1.8	Cold	-0.309	5.83(8)	7.4(3)
		Hot	-0.314	5.8(1)	7.7(4)
1.00 mol/kg NaCl	0.88	Cold	-0.436	10.0(5)	19(3)
		Hot	-0.376	9.8(5)	20(2)
	1.8	Cold	-0.312	5.73(4)	8.1(7)
		Hot	-0.308	5.77(9)	8.3(6)
3.18 mol/kg NaCl	0.88	Cold	-0.283	10.3(1)	27(6)
		Hot	-0.296	10.5(1)	30(7)
	1.8	Cold	-0.207	5.76(3)	9.8(3)
		Hot	-0.228	5.80(3)	8.9(9)

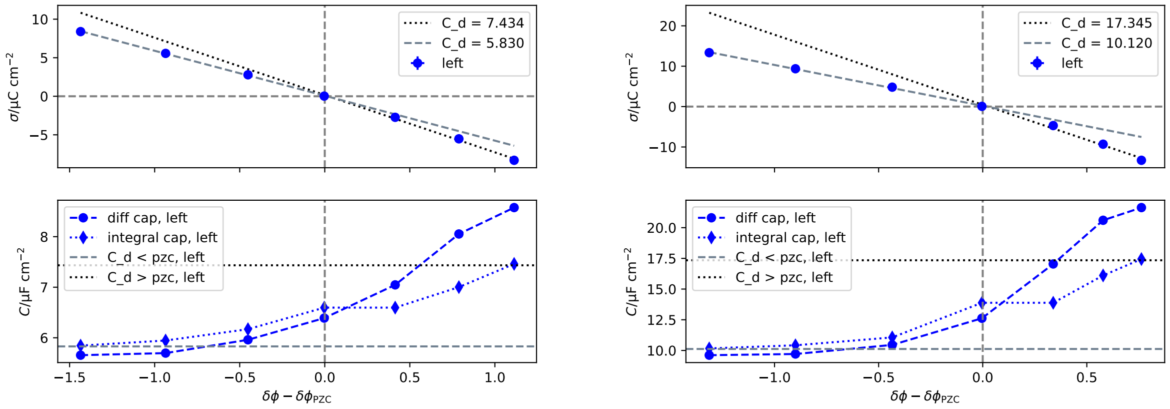

Figure S9: Electrode charge (upper figures) and capacitance (lower figures) as a function of the electrode surface potential, relative to the PZC, for the pure water simulations with parameters 1.81 $\mathring{A}^{-1}$ (left) and 0.88 $\mathring{A}^{-1}$ (right). The dotted and dashed horizontal lines in the capacitance figures represent the fitting indicating the differential capacitance C_d in $\mu F/cm^2$ shown in the upper (charge) figures. In the lower figure, the blue round points are the differential capacitance as calculated using the numerical derivative of the charge with respect to the surface potential and the diamond points represent the integral capacitance, $C_{\text{int}} = \sigma / (\delta\phi - \delta\phi_{\text{pzc}})$

S8 Supporting Information for Electrolyte solutions

S8.1 Summary of Simulation Results

Tables S4a and S4b summarise key results and simulation parameters for the 1 mol/kg NaCl system, for the (a) low and (b) high metallicity electrodes.

Table S4: Results for the 1 mol/kg NaCl system with low and high metallicity electrodes. The columns T_l, T_r and $\Delta\phi$ are the target temperatures for the left and right electrodes, and the target potential difference, respectively. The subscripts l and r refer to the 'left" and "right" electrodes. The column p is the average zz (perpendicular to the electrode plane) component of the pressure tensor. The column J_q is the heat flux resulting from the applied temperature difference, calculated as discussed in section S4. The columns $\delta\phi_\mathrm{l}$ and $\delta\phi_\mathrm{r}$ are differences in potential between the electrode surface and the second minimum in the water density. The columns $R_{\mathrm{K,l}}$ and $R_{\mathrm{K,r}}$ are Kapitza Resistances at the two interfaces. σ represent the average surface charge. The numbers in parentheses indicate the uncertainty (standard error).

T_l/K	T_r/K	$\Delta\phi$/V	p/atm	J_q/GW/m^2	$\delta\phi_\mathrm{l}$/V	$\delta\phi_\mathrm{r}$/V	σ_l/μC/cm^2	σ_r/μC/cm^2	$R_{\mathrm{K,l}}$/Km^2/GW	$R_{\mathrm{K,r}}$/Km^2/GW	ΔT_l/K	ΔT_r/K
250	350	-3.000	−102(6)	6.94(3)	−2.17(1)	0.974(8)	10.66(3)	−10.66(3)	4.25(7)	4.45(8)	29.5(5)	30.9(5)
250	350	-2.000	−107(4)	6.60(3)	−1.49(1)	0.612(7)	6.85(3)	−6.85(3)	4.61(6)	4.7(1)	30.4(4)	31.2(8)
250	350	-1.000	−115(6)	6.39(4)	−0.884(9)	0.183(9)	3.31(2)	−3.31(2)	4.88(6)	5.10(7)	31.2(3)	32.6(5)
250	350	0.000	−588(7)	6.00(3)	−0.318(9)	−0.302(9)	0.04(2)	−0.04(2)	5.03(7)	5.48(8)	30.2(4)	32.9(5)
250	350	1.000	−113(7)	6.35(5)	0.207(8)	−0.851(7)	−3.36(2)	3.36(2)	4.61(8)	5.26(8)	29.2(4)	33.4(6)
250	350	2.000	−601(4)	6.27(4)	0.630(9)	−1.469(7)	−6.84(2)	6.84(2)	4.38(4)	5.24(6)	27.5(2)	32.8(3)
250	350	3.000	−562(5)	6.46(5)	1.002(8)	−2.17(1)	−10.81(2)	10.81(2)	4.4(1)	4.83(8)	28.2(6)	31.2(5)

(a) $\eta = 1.81\,\text{Å}^{-1}$

T_l/K	T_r/K	$\Delta\phi$/V	p/atm	J_q/GW/m^2	$\delta\phi_\mathrm{l}$/V	$\delta\phi_\mathrm{r}$/V	σ_l/μC/cm^2	σ_r/μC/cm^2	$R_{\mathrm{K,l}}$/Km^2/GW	$R_{\mathrm{K,r}}$/Km^2/GW	ΔT_l/K	ΔT_r/K
250	350	-3.000	−181(7)	7.85(9)	−2.37	0.603	19.5	−19.5	3.4(1)	3.67(7)	26.9(5)	28.8(7)
250	350	-2.000	−104(8)	7.15(5)	−1.65(8)	0.35(3)	13.4(6)	−13.4(6)	4.0(1)	4.05(9)	28.3(7)	28.9(5)
250	350	-1.000	−58(5)	6.6(1)	−0.98(5)	0.056(4)	6.5(3)	−6.5(3)	4.7(1)	4.8(2)	31.0(4)	32(1)
250	350	0.000	−478(7)	6.20(8)	−0.44371(3)	−0.368(7)	0.3(2)	−0.3(2)	4.8(1)	5.2(1)	29.5(7)	32.0(5)
250	350	1.000	−79(6)	6.7(1)	0.13(2)	−0.97(1)	−6.7(1)	6.7(1)	4.23(9)	5.04(9)	28.2(2)	33.5(1)
250	350	2.000	−454(4)	6.76(5)	0.368	−1.66	−13.5	13.5	3.8(1)	4.4(1)	26.0(7)	29.7(9)
250	350	3.000	−481(5)	7.47(6)	0.597	−2.38	−19.6	19.6	3.29(9)	3.6(2)	24.6(8)	27(1)

(b) $\eta = 0.88\,\text{Å}^{-1}$

Tables S5a and S5b summarise key results and simulation parameters for the 3.18 mol/kg NaCl system, for the low and high metallicity electrodes, respectively.

S8.2 Temperature, Density and Potential Profiles

Figures S10 and S11 present the interfacial density profiles of oxygen and hydrogen atoms for the 1.0 mol/kg and 3.18 mol/kg systems, respectively, with low metallicity electrodes.

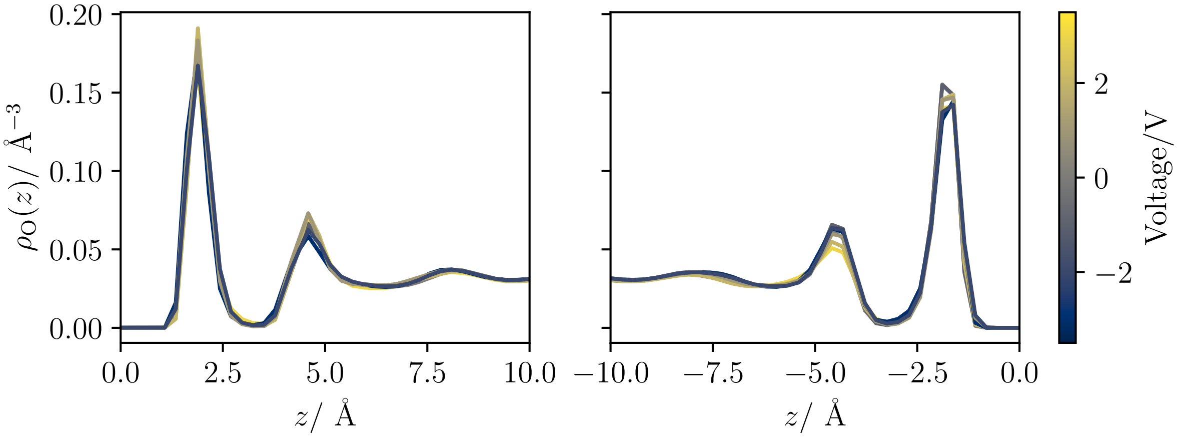

(a) O

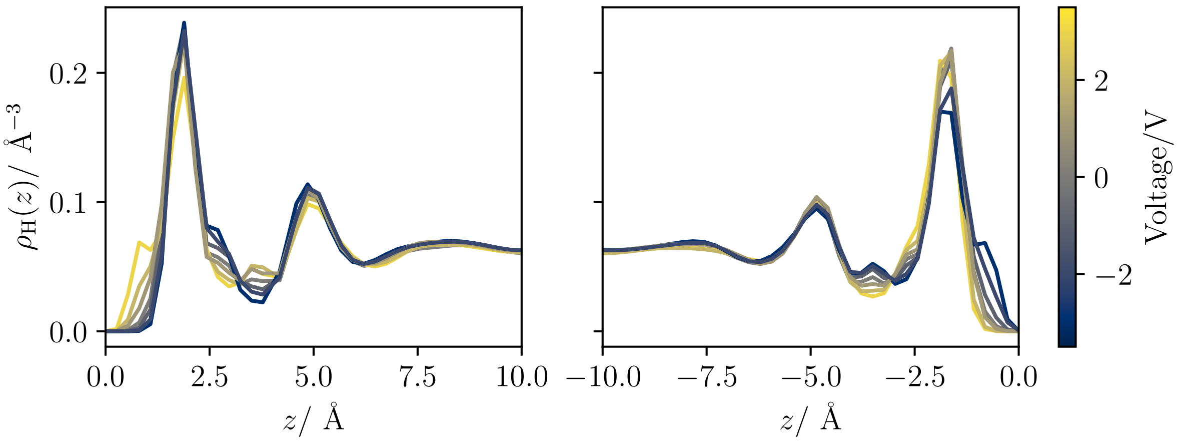

(b) H

Figure S10: Density profiles of the water (a) Oxygen (b) Hydrogen atoms for the 1.00 mol/kg NaCl system for several applied voltages and $(T_{\mathrm{low}}, T_{\mathrm{high}}) = (250\,\mathrm{K}, 350\,\mathrm{K})$. The profiles for the ions are shown in the main text. The line colour indicates the voltage applied between the electrodes. A positive voltage corresponds to the left electrode being negatively polarized and the right electrode positively so. All the results were obtained with $\eta = 1.81\,\text{Å}^{-1}$.

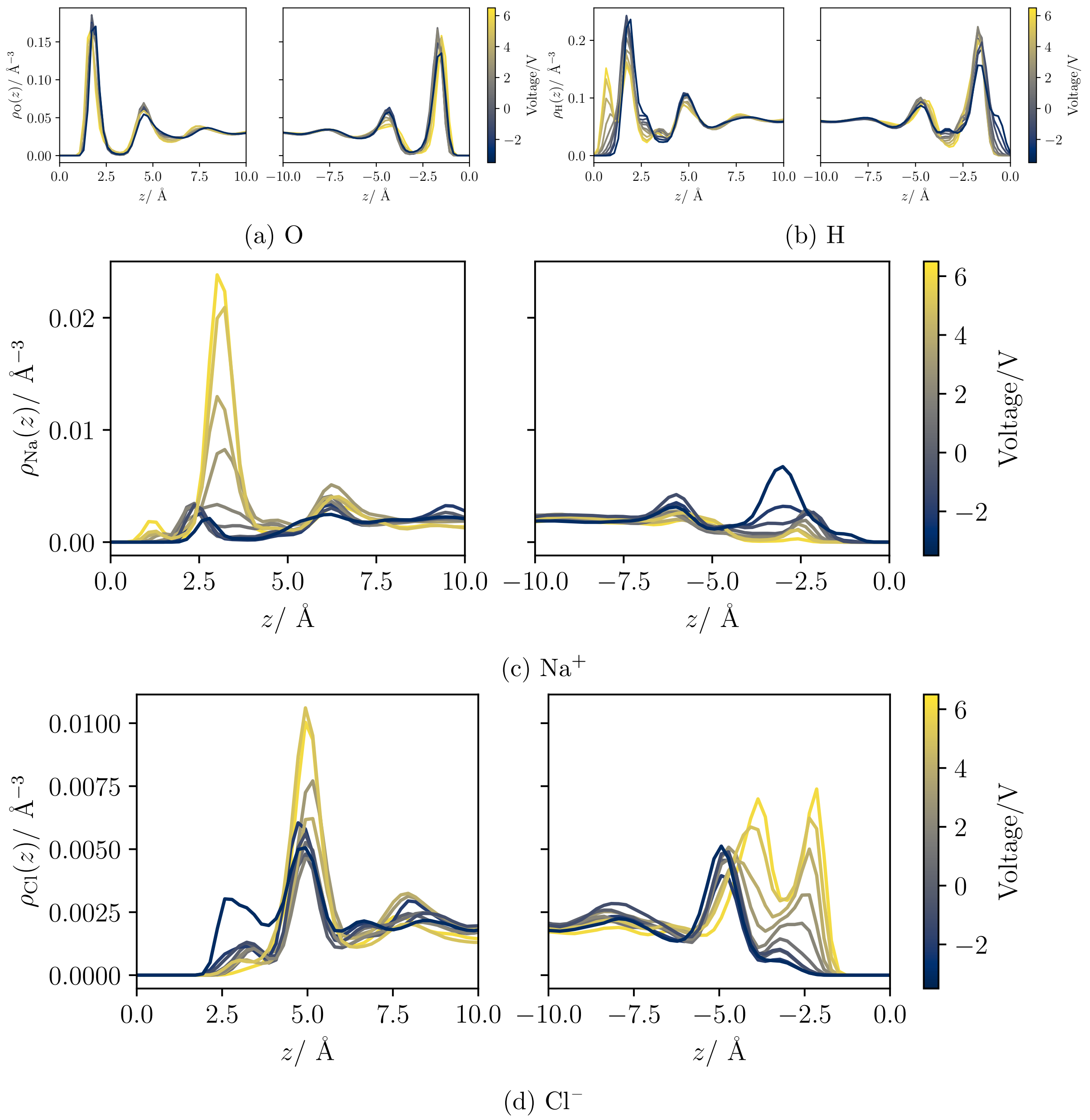

Figure S11: Density profiles of the water (a) Oxygen (b) Hydrogen atoms and ions (c) Na^+ and (d) Cl^- in the 3.18 mol/kg NaCl system for several applied voltages and $(T_{low}, T_{high}) = (250\,K, 350\,K)$. The line colour indicates the voltage applied between the electrodes. A positive voltage corresponds to the left electrode being negatively polarized and the right electrode positively so. All the results were obtained with $\eta = 1.81\,\text{Å}^{-1}$.

Table S5: Same as Table S4 for the 3.18 mol/kg NaCl.

T_l/K	T_r/K	$\Delta\phi$/V	p/atm	J_q/GW/m^2	$\delta\phi_l$/V	$\delta\phi_r$/V	σ_l/μC/cm^2	σ_r/μC/cm^2	$R_{K,l}$/Km^2/GW	$R_{K,r}$/Km^2/GW	ΔT_l/K	ΔT_r/K
250	350	-3	$-1.0(2) \times 10^2$	6.31(5)	−2.13(1)	1.00(1)	11.04(4)	−11.04(4)	4.4(1)	4.5(1)	27.5(5)	28.4(6)
250	350	-2	$-6(1) \times 10^1$	6.07(6)	−1.42(2)	0.66(2)	7.02(3)	−7.02(3)	4.65(5)	4.7(1)	28.2(4)	28.7(4)
250	350	-1	$-1.6(4) \times 10^2$	5.86(3)	−0.82(2)	0.30(2)	3.47(4)	−3.47(4)	4.79(5)	5.14(8)	28.1(3)	30.1(3)
250	350	0	$-1.2(3) \times 10^2$	5.78(4)	−0.22(2)	−0.22(1)	0.06(2)	−0.06(2)	4.85(5)	5.30(7)	28.0(2)	30.6(4)
250	350	1	$-1.8(1) \times 10^2$	5.77(2)	0.266(8)	−0.79(1)	−3.32(3)	3.32(3)	4.65(9)	5.44(6)	26.8(5)	31.4(4)
250	350	2	$-1.6(3) \times 10^2$	5.96(1)	0.68(1)	−1.41(1)	−6.95(2)	6.95(2)	4.3(1)	5.26(6)	25.8(6)	31.3(3)
250	350	3	$-1.1(4) \times 10^2$	6.24(8)	1.07(2)	−2.09(2)	−10.98(3)	10.98(3)	4.2(1)	4.76(6)	26.0(7)	29.7(4)
250	350	4	$-2(1) \times 10^1$	6.58(4)	1.41(1)	−2.792(8)	−14.96(4)	14.96(4)	3.75(9)	4.30(7)	24.7(7)	28.3(3)
250	350	5	−80(9)	6.70(7)	1.76(3)	−3.43(2)	−18.81(5)	18.81(5)	3.6(1)	3.89(7)	24.3(5)	26.0(3)
250	350	6	$-8(1) \times 10^1$	7.11(4)	2.12(2)	−4.08(2)	−22.27(3)	22.27(3)	3.27(2)	3.48(3)	23.3(2)	24.7(3)

(a) $\eta = 1.81\,\text{Å}^{-1}$

T_l/K	T_r/K	$\Delta\phi$/V	p/atm	J_q/GW/m^2	$\delta\phi_l$/V	$\delta\phi_r$/V	σ_l/μC/cm^2	σ_r/μC/cm^2	$R_{K,l}$/Km^2/GW	$R_{K,r}$/Km^2/GW	ΔT_l/K	ΔT_r/K
250	350	-3	$-1.1(1) \times 10^2$	7.41(5)	−2.537(7)	0.43(1)	23.20(6)	−23.20(6)	3.03(7)	3.39(7)	22.5(6)	25.1(4)
250	350	-2	−72(4)	6.65(3)	−1.74(2)	0.31(2)	15.37(4)	−15.37(4)	3.89(4)	4.19(9)	25.9(2)	27.9(5)
250	350	-1	$-5(1) \times 10^1$	6.15(3)	−0.980(8)	0.13(1)	7.33(3)	−7.33(3)	4.56(6)	4.69(9)	28.1(4)	28.9(6)
250	350	0	−50(9)	5.86(5)	−0.29(2)	−0.29(1)	0.10(3)	−0.10(3)	4.83(6)	5.27(9)	28.3(2)	30.9(3)
250	350	1	−70(8)	6.19(3)	0.16(1)	−0.95(1)	−7.08(7)	7.08(7)	4.22(7)	4.952(7)	26.2(5)	30.7(1)
250	350	2	−114(6)	6.56(4)	0.39(2)	−1.703(6)	−15.15(8)	15.15(8)	3.67(7)	4.32(5)	24.1(5)	28.3(3)
250	350	3	$-1.0(2) \times 10^2$	7.27(2)	0.50(3)	−2.47(1)	−22.8(1)	22.8(1)	3.11(2)	3.51(3)	22.6(1)	25.5(1)

(b) $\eta = 0.88\,\text{Å}^{-1}$

Figure S12 presents the potential profile (calculated from the charge density using Equation (9)) for the 1 mol/kg system with the low-metallicity electrodes.

Figure S13 presents examples of the temperature profiles in the salt systems, 1.00 mol/kg and 3.18 mol/kg with high metallicity electrodes under applied voltages of 1 V.

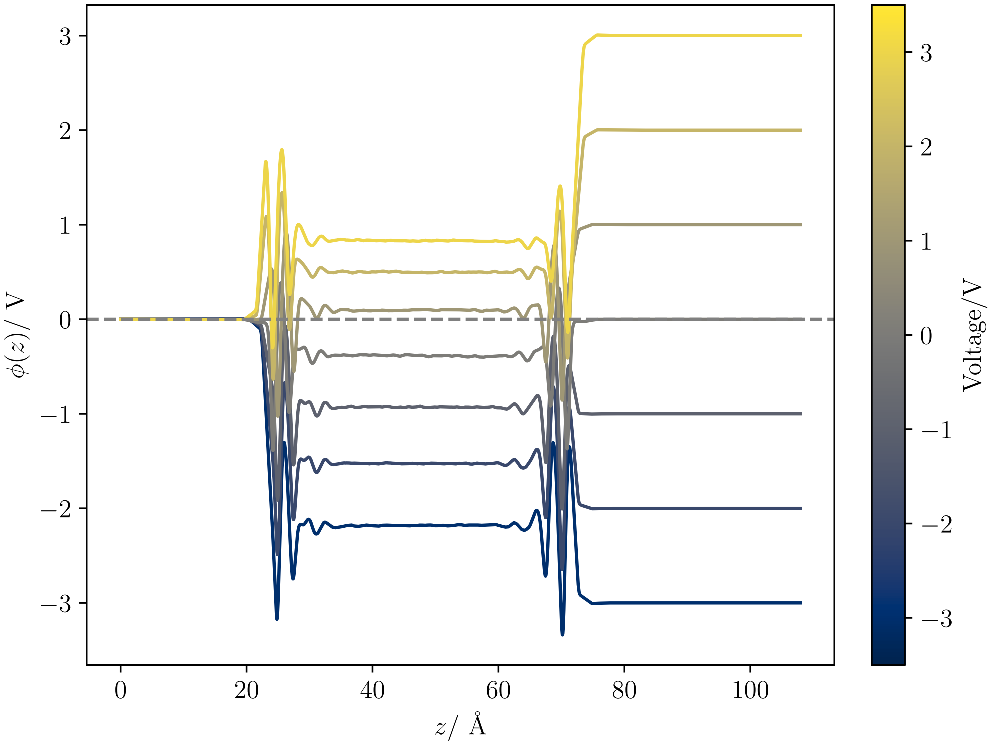

Figure S12: The potential profiles in the 1.00 mol/kg NaCl system for several applied voltages and $(T_{\rm low}, T_{\rm high}) = (250\,\mathrm{K}, 350\,\mathrm{K})$. The calculations were performed with low metallicity electrodes.

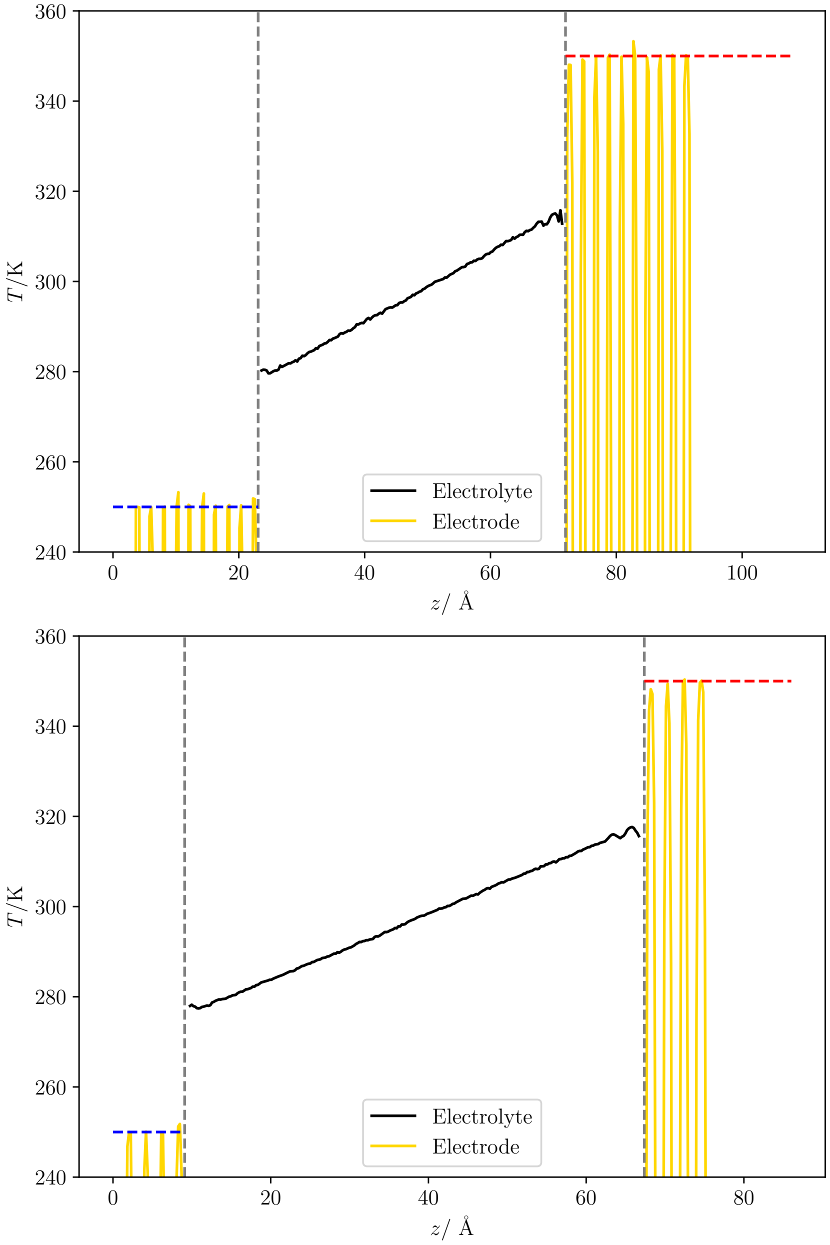

Figure S13: Example temperature profiles for the 1.00 mol/kg (top) and 3.18 mol/kg (bottom) systems each with an applied potential difference of 1.0 V and electrode temperatures of $(T_{\mathrm{low}}, T_{\mathrm{high}}) = (250\,\mathrm{K}, 350\,\mathrm{K})$. The results were obtained with high metallicity electrodes ($\eta = 0.88\,\mathrm{\AA}^{-1}$). The yellow lines represent the electrode temperatures and the black line the electrolyte temperature (averaged over all non-electrode atoms). The dashed horizontal lines indicate the target thermostat temperatures and the dashed vertical lines the locations of the electrode interface.

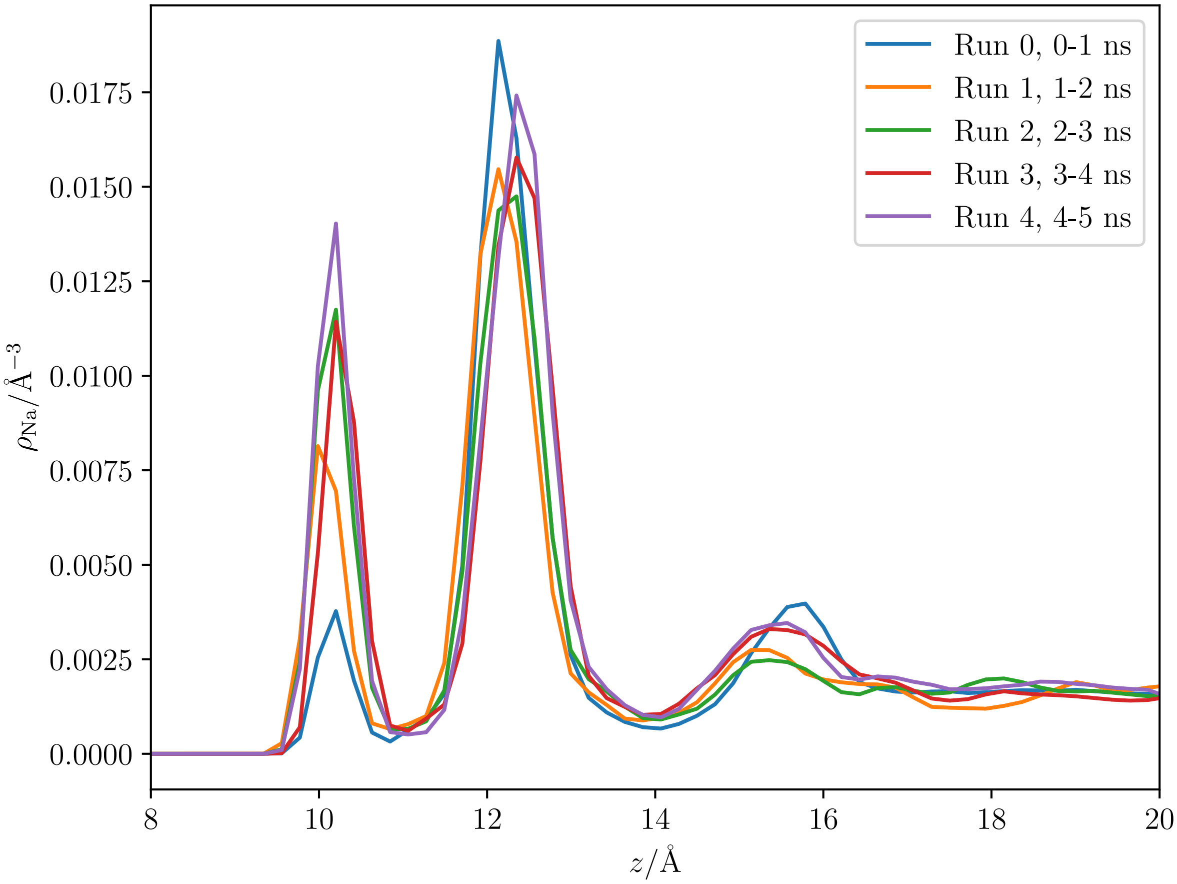

Figure S14: Evolution of the sodium ion density profile from the negatively polarized electrode in the high-metallicity ($\eta = 0.88\,\mathrm{\AA}^{-1}$) 3.18 mol/kg-NaCl system under 3V, over time. Each line represents the average density profile over consecutive 1 ns simulation runs.

S9 Vibrational Density of States Analysis

The interfacial Vibrational Density of States (VDOS) was computed for the water system under 0.0 V and 3.0 V. We calculated the Fourier transform of the normalised Velocity Auto-Correlation Function (VACF),

$$D_\alpha(f) = \int_0^\infty \frac{C_{\alpha,z}(t)}{C_{\alpha,z}(0)} \exp(-2\pi i f t) dt, \tag{10}$$

where the VACF of atoms of type α,

$$C_{\alpha,z}(t) = \left\langle \sum_{i \in \alpha} v_{i,z}(t) \cdot v_{i,z}(0) \right\rangle, \tag{11}$$

where the summation is over all atoms i with the type α. Note that we have used the component of the velocity parallel to the heat flux (z) to compute the VACF.

Extra simulations were performed with a reduced 0.5 fs timestep, in order to capture as much short timescale resolution. To approximate an ensemble average, 61 independent replicas were run, each 5 ps long, to focus on the short-time dynamics. The replicas were made statistically independent by using different random seeds for each thermostat and running the simulations for a random number of time steps between 1000 and 6000 before recording production data. Only atoms in the interfacial region were considered for the VACF. Oxygen and hydrogen atoms initially within 4 Å from each electrode surface were taken to be the interfacial region for purposes of the VACF calculations. By the end of these short 5 ps simulations, approximately 89 % of atoms in this region remained within the first 4 Å from the surface and 99 % within the first 6 Å (approximately the location of the second water layer peak). The average displacement, across all simulations, from the interface for the surface water atoms was 2.3 Å for the cold electrode at 0 V. All electrode atoms within the first atomic layer were used to calculate the electrode VACFs. As shown in S15, there is little difference between the VDOS between the zero electrostatic potential and 3 V. The

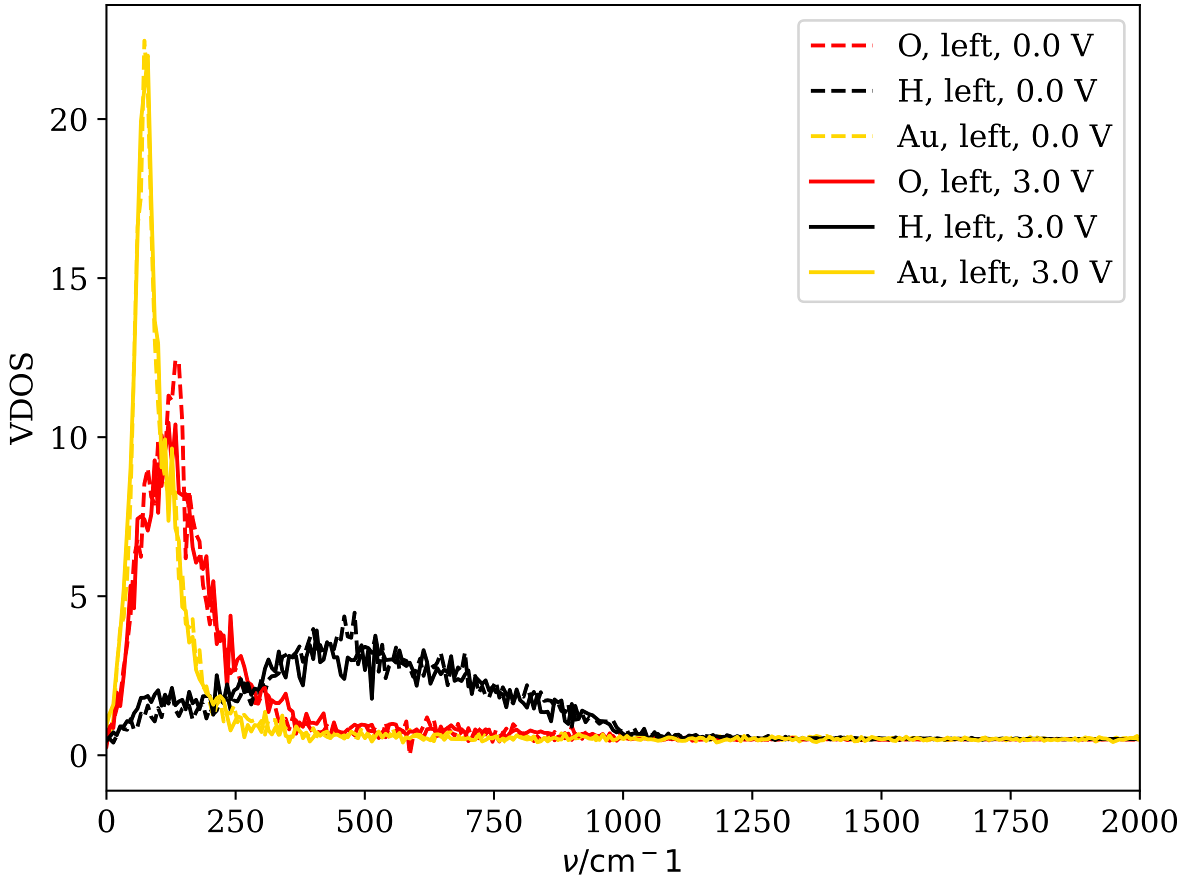

Figure S15: The real component of the VDOS of the cold (250 K) electrode interface in the water system, at 0.0 V (dashed line) and 3.0 V (solid line). Each line colour corresponds to a different component of the system — oxygen (red), hydrogen (black) and metal (yellow) atoms. The calculations were performed with the low metallicity electrode.

results were obtained for the cold electrode. The hot electrode showed a similar insensitivity to applied potential.

S10 Interfacial Orientation Distributions

The probability distribution of the orientation of the water molecules provides powerful insight into the interfacial structure. We calculated the orientation distributions of the interfacial water molecules at each electrode surface. Water molecules were selected if their oxygen atoms were located within 4 Å from the electrode surface. The molecular orientations were binned from 100 frames of each simulation trajectory. Simulation replicas for the same applied voltage were accumulated into the same histogram. The histograms were normalised into density plots. The orientation angle was calculated between the water dipole moment vector and the interface normal (the z and $-z$ directions for the left and right interfaces, respectively).

For all systems considered here, there are two major peaks in the orientation distributions: a low-angle peak around 60°, representing molecules with the oxygen atom pointing towards the surface, and a peak at ≈90°–100°, representing water molecules with their dipole moment close to perpendicular to the electrode surface, with a slight preference for hydrogen to point toward the surface. The location of the high-angle peak varies with voltage and depends on the interface and electrolyte being considered. At high negative polarizations of the high metallicity interface in contact with $NaCl_{aq}$, the peaks reached 133°, which corresponds to the dipole moment vector with hydrogens oriented towards the surface.

Figures S16a and S16b show the orientational distributions at the interfaces of the low metallic water system, $\eta = 1.81\,\text{Å}^{-1}$. For highly negative voltages, there is a significant population at $\approx 100°$. This indicates the molecules have their hydrogen atoms pointing toward the surface. As the voltage becomes more negative, this peak decreases to $\approx 95°$, indicating the molecules are lying perpendicular to the interface. A second population appear around $\approx 56°$, increasing with positive surface polarization. This second peak indicates molecules with the oxygen atom pointing towards the interface. The high-angle peak is dominant in positive voltages.

Figures S16c and S16d show the orientational histograms for the high metallicity electrode

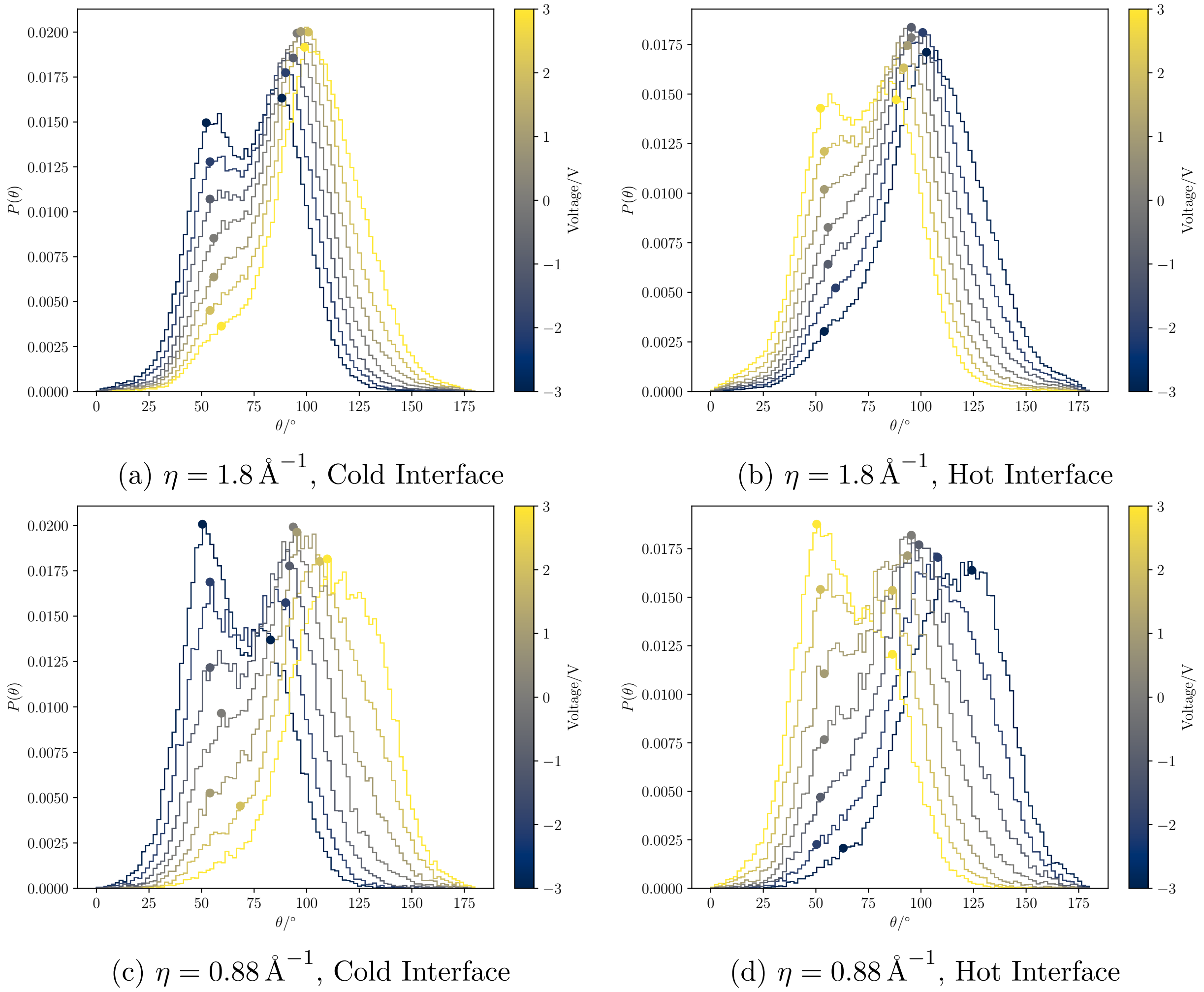

(a) $\eta = 1.8\,\text{Å}^{-1}$, Cold Interface

(b) $\eta = 1.8\,\text{Å}^{-1}$, Hot Interface

(c) $\eta = 0.88\,\text{Å}^{-1}$, Cold Interface

(d) $\eta = 0.88\,\text{Å}^{-1}$, Hot Interface

Figure S16: Normalised probability distributions of the HOH angle bisector orientation in the first water layer of the pure water system. The y-axis represents the probability density. The upper plots (a. and b.) show the orientation of the system with the large η parameter (less metallic surface), whilst the lower plots (c. and d.) show the system with the smaller η parameter (more metallic surface). The plots on the left (a. and c.) and right (b. and d.) show the distributions for the cold and hot interfaces, respectively. Each line is coloured according to the potential difference between the two electrodes, $\Delta\phi = \phi_{\text{right}} - \phi_{\text{left}}$.

and water system, $\eta = 0.88\,\text{Å}^{-1}$. Unlike the low-metallicity electrode, there is only a single population ($\sim 50°$ for positive voltages and $\approx 120°$ for negative voltages). The higher image charges of the more metallic electrode induce a stronger polarization of the interfacial water molecules, with the hydrogens preferentially oriented towards the electrodes.

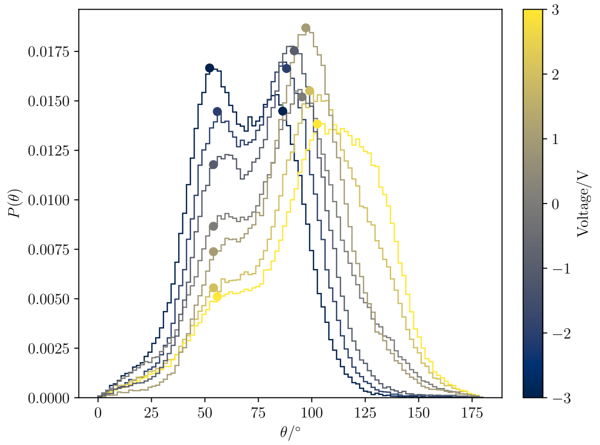

(a) $\eta = 1.8\,\text{Å}^{-1}$, Cold Interface

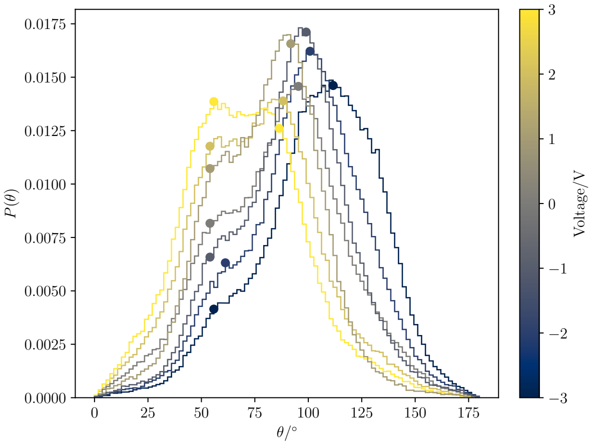

(b) $\eta = 1.8\,\text{Å}^{-1}$, Hot Interface

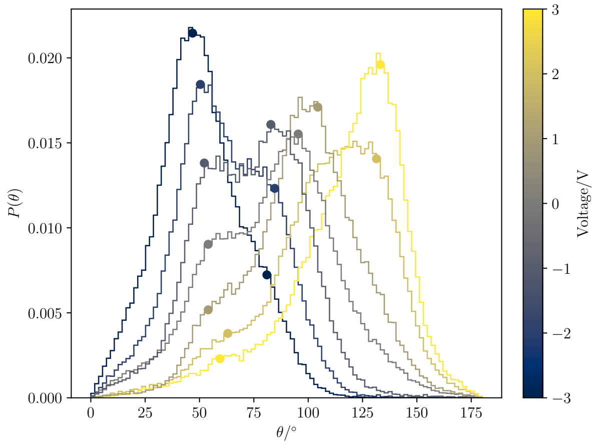

(c) $\eta = 0.88\,\text{Å}^{-1}$, Cold Interface

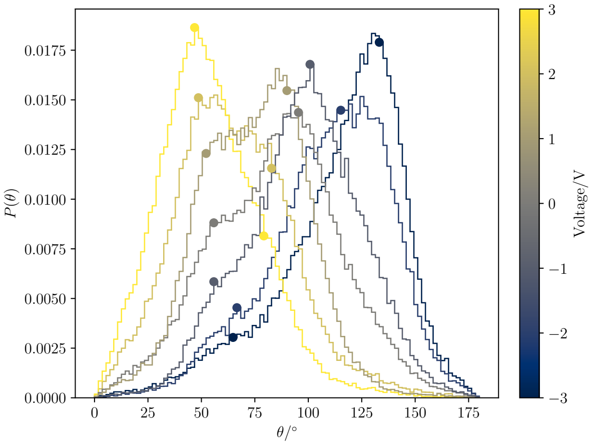

(d) $\eta = 0.88\,\text{Å}^{-1}$, Hot Interface

Figure S17: Normalized probability distributions of the HOH angle bisector orientation in the first water layer of the 1 mol/kg NaCl system. The y-axis represents the probability density. The upper plots (a. and b.) show the orientation of the system with the large η parameter (less metallic surface), whilst the lower plots (c. and d.) show the system with the smaller η parameter (more metallic surface). The plots on the left (a. and c.) and right (b. and d.) show the distributions for the cold and hot interfaces, respectively. Each line is coloured according to the potential difference between the two electrodes, $\Delta\phi = \phi_{\text{right}} - \phi_{\text{left}}$.

The addition of salt has a significant effect on the water orientational distributions (Figure S17). In the low metallicity systems (Figures S17a and S17b), there is an enhancement in

the right-most orientation peak as the voltage gets closer to zero, in contrast to pure water, where the peak monotonically decreases in height. Above zero volts, this peak decreases in height, much like in the water system; however, there is still a significant population at 3 V with a peak at 90°. The left-most peak increases in height at more positive voltages, indicating that the water becomes strongly oriented with oxygen pointing towards the interface. Interestingly, here, there is less symmetry between the cold and hot interface; the hot interface (Figure S17b) does not show the same clear bimodal distribution as the cold interface, showing instead two merged peaks, due to a significant left shifting of the high-angle peak.

The salt system with high metallicity electrode ($\eta = 0.88\,\text{Å}^{-1}$), by contrast, shows only a single maxima for all voltages explored, for both the hot and cold interfaces (Figures S17c and S17d), much like the highly metallic water system. The only exception is the presence of a shoulder at 0 V. This single peak migrates leftwards with increasingly positively polarized interfaces, from $\approx 130°$ at -3.0 V to $\approx 30°$ at 3.0 V at the hot interface.

References

(S1) Heinz, H.; Vaia, R. A.; Farmer, B. L.; Naik, R. R. Accurate Simulation of Surfaces and Interfaces of Face- Centered Cubic Metals Using 12-6 and 9-6 Lennard- Jones Potentials. *The Journal of Physical Chemistry C* **2008**, *112*, 17281–17290.

(S2) Berendsen, H. J. C.; Grigera, J. R.; Straatsma, T. P. The missing term in effective pair potentials. *The Journal of Physical Chemistry* **1987**, *91*, 6269–6271.

(S3) Ryckaert, J.-P.; Ciccotti, G.; Berendsen, H. J. C. Numerical Integration of the Cartesian Equations of Motion of a System with Constraints: Molecular Dynamics of *n*-Alkanes. *Journal of Computational Physics* **1977**, *23*, 327–341.

(S4) Dang, L. X. Mechanism and Thermodynamics of Ion Selectivity in Aqueous Solu-

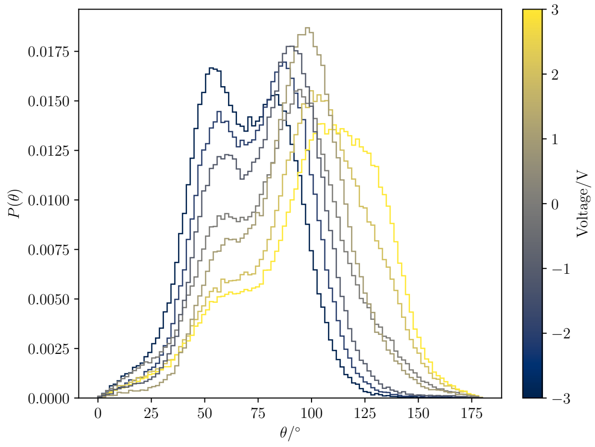

(a) $\eta = 1.8\,\text{Å}^{-1}$, Cold Interface

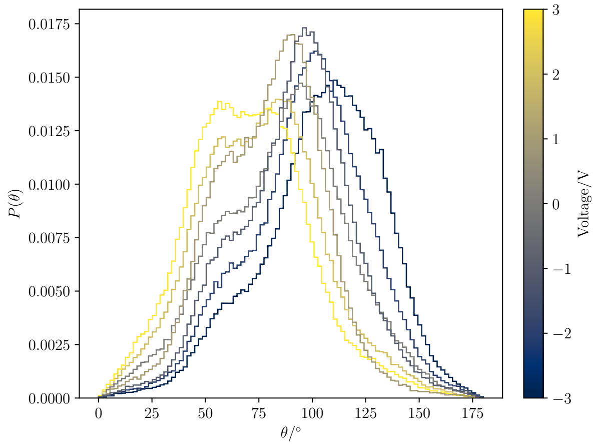

(b) $\eta = 1.8\,\text{Å}^{-1}$, Hot Interface

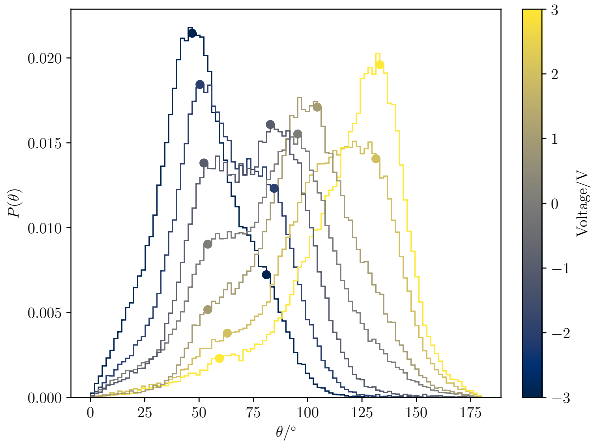

(c) $\eta = 0.88\,\text{Å}^{-1}$, Cold Interface

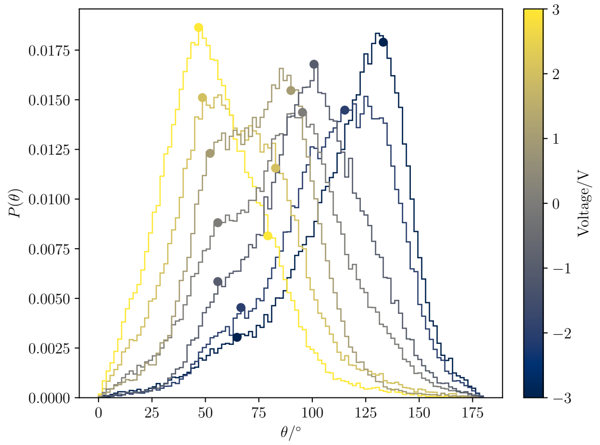

(d) $\eta = 0.88\,\text{Å}^{-1}$, Hot Interface

Figure S18: Distributions of the HOH angle bisector orientation in the first water layer of the 3.18 mol/kg NaCl system. The y-axis represents the density of observations. The upper plots (a. and b.) show the orientation of the system with the large η parameter (less metallic surface), whilst the lower plots (c. and d.) show the system with the smaller η parameter (more metallic surface). The plots on the left (a. and c.) and right (b. and d.) show the distributions for the cold and hot interfaces, respectively. Each line is coloured according to the potential difference between the two electrodes, $\Delta\phi = \phi_{\text{right}} - \phi_{\text{left}}$.

tions of 18-Crown-6 Ether: A Molecular Dynamics Study. *Journal of the American Chemical Society* **1995**, *117*, 6954–6960.

(S5) Serva, A.; Scalfi, L.; Rotenberg, B.; Salanne, M. Effect of the metallicity on the capacitance of gold–aqueous sodium chloride interfaces. *The Journal of Chemical Physics* **2021**, *155*, 044703.

(S6) Ahrens-Iwers, L. J. V.; Janssen, M.; Tee, S. R.; Meißner, R. H. ELECTRODE: An electrochemistry package for atomistic simulations. *The Journal of Chemical Physics* **2022**, *157*, 084801.

(S7) Hockney, R. W.; Eastwood, J. W. *Computer Simulation Using Particles*; McGraw-Hill, 1988.

(S8) Ahrens-Iwers, L. J. V.; Meißner, R. H. Constant Potential Simulations on a Mesh. *The Journal of Chemical Physics* **2021**, *155*, 104104.

(S9) Dufils, T.; Jeanmairet, G.; Rotenberg, B.; Sprik, M.; Salanne, M. Simulating Electrochemical Systems by Combining the Finite Field Method with a Constant Potential Electrode. *Physical Review Letters* **2019**, *123*, 195501, Publisher: American Physical Society.

(S10) Thompson, A. P.; Aktulga, H. M.; Berger, R.; Bolintineanu, D. S.; Brown, W. M.; Crozier, P. S.; In 'T Veld, P. J.; Kohlmeyer, A.; Moore, S. G.; Nguyen, T. D.; Shan, R.; Stevens, M. J.; Tranchida, J.; Trott, C.; Plimpton, S. J. LAMMPS - a flexible simulation tool for particle-based materials modeling at the atomic, meso, and continuum scales. *Computer Physics Communications* **2022**, *271*, 108171.

(S11) Reed, S. K.; Lanning, O. J.; Madden, P. A. Electrochemical interface between an ionic liquid and a model metallic electrode. *The Journal of Chemical Physics* **2007**, *126*, 084704.

(S12) Tee, S. R.; Searles, D. J. Fully periodic, computationally efficient constant potential molecular dynamics simulations of ionic liquid supercapacitors. *The Journal of Chemical Physics* **2022**, *156*, 184101.

(S13) Shewchuk, J. *An Introduction to the Conjugate Gradient Method Without the Agonizing Pain*; Carnegie-Mellon University. Department of Computer Science, 1994.

(S14) Gingrich, T. Simulating Surface Charge Effects in Carbon Nanotube Templated Ionic Crystal Growth. **2010**,

(S15) `https://github.com/bresmegroup/electrode-paper`, 2025; [Accessed 30-09-2025].

(S16) Olarte-Plata, J. D.; Bresme, F. Thermal conductance of the water–gold interface: The impact of the treatment of surface polarization in non-equilibrium molecular simulations. *The Journal of Chemical Physics* **2022**, *156*, 204701.

(S17) Stukowski, A. Visualization and analysis of atomistic simulation data with OVITO –the Open Visualization Tool. *Modelling and Simulation in Materials Science and Engineering* **2010**, *18*, 015012.
